# Genome-wide CRISPR/Cas9 screen identifies SLC39A9 and PIK3C3 as crucial entry factors for Ebola virus infection

**DOI:** 10.1371/journal.ppat.1012444

**Published:** 2024-08-22

**Authors:** Mingli Gong, Cheng Peng, Chen Yang, Zhenhua Wang, Hongwu Qian, Xue Hu, Peng Zhou, Chao Shan, Qiang Ding

**Affiliations:** 1 School of Basic Medical Sciences, Tsinghua University, Beijing, China; 2 State Key Laboratory of Virology, Wuhan Institute of Virology, Center for Biosafety Mega-Science, Chinese Academy of Sciences, Wuhan, Hubei, China; 3 The First Affiliated Hospital of USTC, MOE Key Laboratory for Membraneless Organelles and Cellular Dynamics, Hefei National Research Center for Interdisciplinary Sciences at the Microscale, Division of Life Sciences and Medicine, University of Science and Technology of China, Hefei, China; 4 CAS Key Laboratory of Special Pathogens, Wuhan Institute of Virology, Center for Biosafety Mega-Science, Chinese Academy of Sciences, Wuhan, Hubei, China; 5 SXMU-Tsinghua Collaborative Innovation Center for Frontier Medicine, Shanxi Medical University, Taiyuan, China; University of Texas Medical Branch / Galveston National Laboratory, UNITED STATES OF AMERICA

## Abstract

The Ebola virus (EBOV) has emerged as a significant global health concern, notably during the 2013–2016 outbreak in West Africa. Despite the clinical approval of two EBOV antibody drugs, there is an urgent need for more diverse and effective antiviral drugs, along with comprehensive understanding of viral-host interactions. In this study, we harnessed a biologically contained EBOVΔVP30-EGFP cell culture model which could recapitulate the entire viral life cycle, to conduct a genome-wide CRISPR/Cas9 screen. Through this, we identified PIK3C3 (phosphatidylinositide 3-kinase) and SLC39A9 (zinc transporter) as crucial host factors for EBOV infection. Genetic depletion of SLC39A9 and PIK3C3 lead to reduction of EBOV entry, but not impact viral genome replication, suggesting that SLC39A9 and PIK3C3 act as entry factors, facilitating viral entry into host cells. Moreover, PIK3C3 kinase activity is indispensable for the internalization of EBOV virions, presumably through the regulation of endocytic and autophagic membrane traffic, which has been previously recognized as essential for EBOV internalization. Notably, our study demonstrated that PIK3C3 kinase inhibitor could effectively block EBOV infection, underscoring PIK3C3 as a promising drug target. Furthermore, biochemical analysis showed that recombinant SLC39A9 protein could directly bind viral GP protein, which further promotes the interaction of viral GP protein with cellular receptor NPC1. These findings suggests that SLC39A9 plays dual roles in EBOV entry. Initially, it serves as an attachment factor during the early entry phase by engaging with the viral GP protein. Subsequently, SLC39A9 functions an adaptor protein, facilitating the interaction between virions and the NPC1 receptor during the late entry phase, prior to cathepsin cleavage on the viral GP. In summary, this study offers novel insights into virus-host interactions, contributing valuable information for the development of new therapies against EBOV infection.

## Author summary

Ebola virus (EBOV) remains a significant global health threat. The limited availability of high-containment (BSL-4) facilities hinders our understanding of the interaction of virus with host cells and the development of effective treatments. To address this, we utilized a biologically contained EBOVΔVP30-EGFP model to identify critical host factors necessary for EBOV infection through a genome-wide CRISPR/Cas9 screen conducted at BSL-2 lab. Our study revealed that two proteins, PIK3C3 and SLC39A9, are essential for the virus to enter host cells. We found that PIK3C3 is required for EBOV endosomal trafficking, and inhibiting its kinase activity can significantly reduce infection, making it a promising target for antiviral therapy. Additionally, we discovered that SLC39A9 facilitates viral entry by interacting with the viral glycoprotein (GP) and enhancing its binding to the cell’s NPC1 receptor, rather than through its usual role in zinc transport. These findings offer new insights into how EBOV enters cells and highlight potential targets for developing therapies to combat Ebola virus infection.

## Introduction

Ebola virus (EBOV) was discovered in 1976 and has since posed a public health threat as a BSL-4 pathogen with high pathogenicity, yielding a mortality rate that can reach up to 90% [1,2]. Manifesting symptoms such as severe hemorrhagic fever, headache, myalgia, and bloody diarrhea [3], EBOV poses a substantial risk to human, particularly in West Africa. The resurgence of EBOV as a significant public health concern, notably during the 2013–2016 outbreak in West Africa, resulted in over 11,000 deaths [4,5].

Presently, two vaccines [6,7] and two neutralizing antibodies were approved in 2020 [8,9] for EBOV intervention. However, these interventions face challenges. Despite the high potency of these two neutralizing antibodies targeting EBOV glycoprotein (GP) [10,11], concerns persist regarding potential long-term risks of viral escape and the development of resistance. A promising avenue for overcoming this challenge lies in combining direct antiviral agents with host-targeting agents, as resistance to host-targeted therapeutics remains relatively rare [12–14]. Thus, uncovering novel host factors essential for EBOV infection and gaining a deeper understanding of virus-host interactions are crucial for discovering new therapeutic targets and advancing antiviral drug development.

EBOV is an enveloped virus belonging to the *Filoviridae* family, characterized by a negative-sense single-stranded RNA genome that encodes at least seven structural proteins: nucleoprotein (NP), viral protein (VP) 35, VP40, glycoprotein (GP), VP30, VP24, and an RNA-dependent RNA polymerase (L) [1,15]. During infection, EBOV initially binds to the cell surface, facilitated by viral glycoprotein and various host factors on the cell membrane, including T cell immunoglobulin and mucin domain 1 (TIM-1) [16–20], dendritic cell-specific ICAM-3-grabbing nonintegrin (DC-SIGN) [21,22] and Tyro3 protein kinase (TAM) [23] etc. Once attached, the virus is internalized, primarily through macropinocytosis, into early endosomes [24,25]. Internalized virions progress to late endosomes/lysosomes, where the acidic environment facilitates the cleavage of the mucin domain and glycan cap of GP by host cysteine proteases cathepsin B (CTSB) and cathepsin L (CTSL), generating the primed EBOV GP (GPcl) which exposes the receptor binding domain (RBD) [26–29]. GPcl subsequently interacts with the intracellular receptor Niemann-Pick C1 (NPC1), triggering fusion between viral and cellular membranes. This fusion releases the viral genome into the cytoplasm, initiating viral transcription and replication processes [30–32].

Numerous critical host factors have been identified for EBOV infection using diverse screening strategies. NPC1 was identified as the viral receptor through both a loss-of-function haploid gene-trapping method [30] and a small-molecular inhibitor screen [31]. Additional factors, such as the proteases CTSB and CTSL for GP cleavage [26–29], proteins associated with the homotypic fusion and vacuole protein sorting (HOPS) complex, and PIKFYVE crucial for endosome transport and maturation, were identified using haploid gene-trapping method and shRNA knockdown screen [33]. The GeCKO (genome-wide CRISPR knockout) screen strategy, applied to authentic EBOV in a BSL-4 laboratory [34], underscored the critical role of N-acetylglucosamine-1-phosphate transferase in EBOV entry.

The major hurdle in studying authentic EBOV lies in the necessity of a BSL-4 facility. To identify additional cellular factors essential for EBOV infection, we conducted GeCKO screens utilizing a previously established EBOVΔVP30-EGFP cell culture model, which faithfully recapitulates the entire life cycle of EBOV in BSL-2 laboratory [35,36]. The success of our screens is underscored by the identification of critical host factors which have been well-demonstrated during EBOV infection, including NPC1, TIM-1, HOPS complex proteins, and the UVRAG complex. Based on functional analysis of top-ranking candidates, our focus converged on PIK3C3 and SLC39A9. In this study, we demonstrate the pivotal role of PIK3C3 and SLC39A9 in EBOV infection and unravel the underlying mechanisms of their significance in viral life cycle.

## Results

### GeCKO screens identify host factors critical for EBOV infection

**Fig 1:**
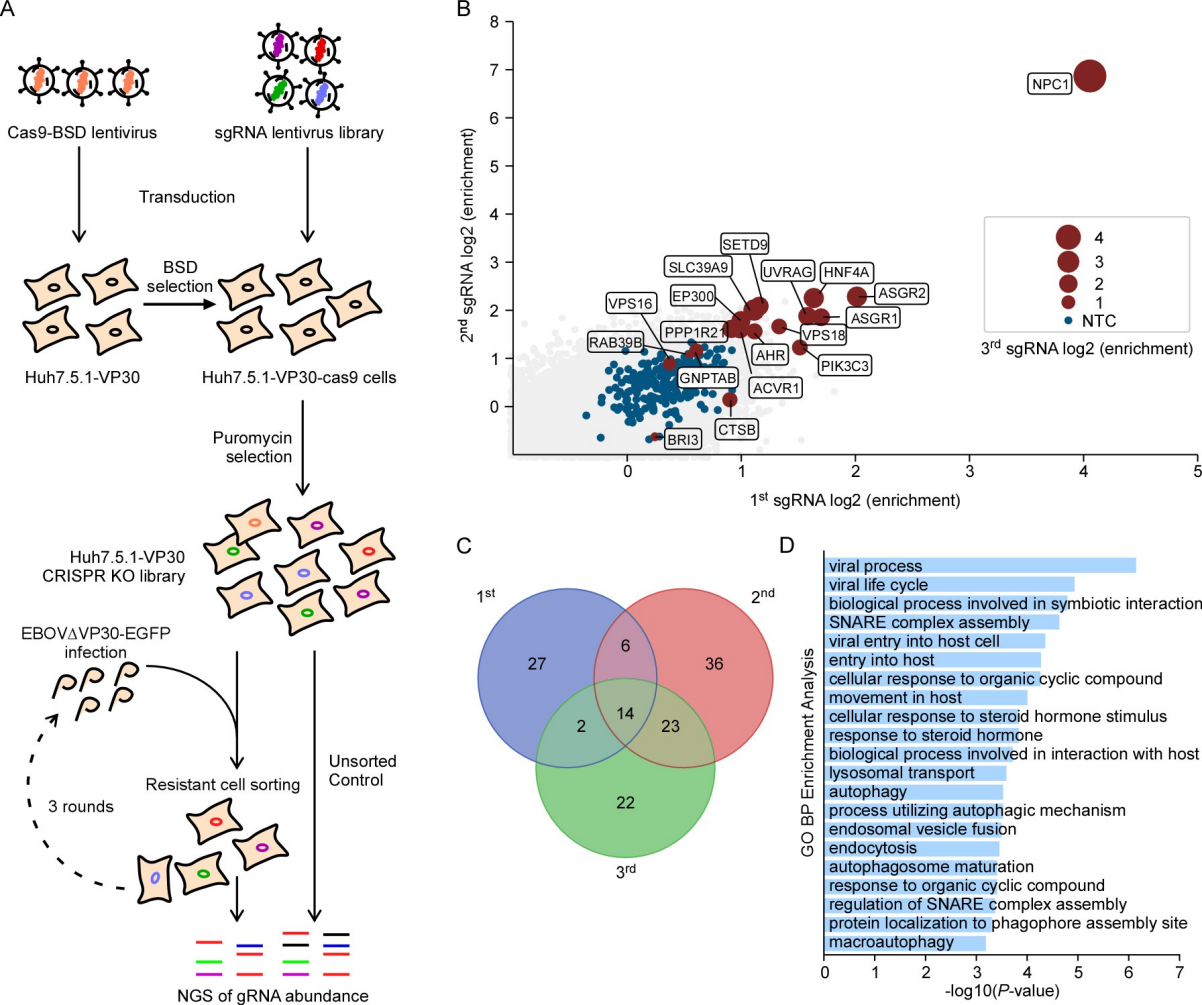
Genome-wide CRISPR/Cas9 KO screen identifies critical host factors for EBOV infection. (**A**) Schematic representation of the genome-wide CRISPR KO screen. Huh7.5.1-VP30-Cas9 CRISPR KO cells were infected with EBOVΔVP30-EGFP at MOI = 1, and GFP-negative cells were sorted at 3 days post-infection (dpi). Three rounds of infection and sorting were performed, and sgRNA abundance was analyzed using next-generation sequencing in both uninfected and selected cell populations. (**B**) Significance of enriched genes from three rounds of CRISPR KO screen based on MAGeCK analysis. The x and y-axes represent the mean log2 fold change of sgRNA counts from the first and second rounds of selection, respectively. Red dots indicate top candidates in the third selection, with circle diameter representing the number of enriched sgRNAs. Non-targeting controls are indicated as blue dots. The top 300 ranked gene list of three selections is provided in **S1 Table**. (**C**) Venn diagram illustrating the overlap of top hits (p value < 0.005, **S1 Table**) from each round of the screens. (**D**) Gene Ontology Biological Process (GO BP) analysis of the top enriched genes from (**C**). The complete list of GO terms is available in **S2 Table**. GO analysis was performed using ToppGene.

To unveil host factors essential for EBOV infection, we conducted genome-wide screens using the CRISPR/Cas9 system [37–39]. Our approach was based on a biologically contained EBOVΔVP30-EGFP cell culture model [35,36], where the viral VP30 gene is replaced by the EGFP reporter gene in the viral genome. This model faithfully recapitulates the entire life cycle of EBOV in human hepatoma Huh7.5.1 cells ectopically expressing viral VP30 (Huh7.5.1-VP30) (**Fig 1A**). To achieve this, we initially transduced Huh7.5.1-VP30 cells with lentivirus carrying Cas9 and blasticidin S deaminase (BSD) genes to generate the Huh7.5.1-VP30-Cas9 cells after blasticidin selection. Then, we transduced the Huh7.5.1-VP30-Cas9 cells with a genome-wide lentiviral CRISPR-Cas9 knockout (KO) Brunello library [37,40]. Following puromycin selection, the CRISPR KO library cells were inoculated with EBOVΔVP30-EGFP virus (MOI = 1), such that virtually all cells expressed the EGFP after 3 days of infection (**S1 Fig**). Cells lacking EGFP expression were sorted, propagated and then re-inoculated with EBOV-ΔVP30-EGFP (**Figs 1A and S1**). After three rounds of infection and sorting, genomic DNA from EGFP-negative cells was harvested, sgRNAs were sequenced, and analyzed using MAGeCK [41] (**Fig 1A and 1B and S1 Table**). NPC1, the cellular receptor of EBOV [30,31], consistently ranked as the top candidate. Additionally, numerous known host factors critical for EBOV infection were also significantly enriched, including HOPS complex proteins [42], UVRAG (Ultraviolet Radiation Resistance-Associated) [42,43], TIM-1 [16–20], GNPTAB [34], CTSB [26–29] (**Fig 1B and S1 Table**). Those top enriched host factors (p value < 0.05) in three rounds of selection were often observed in at least two selections **(Fig 1C and S2 Table)**. Gene ontology (GO) analysis of those top enriched genes (n = 45) revealed many biological processes related to viral infection, such as the viral life cycle, viral entry into host cells, lysosome transport, and endosomal vesicle fusion, many of which are highly relevant to EBOV infection, especially the entry process **(Fig 1D and S2 Table)**.

Taken together, the identification of known critical host factors and biological processes linked to EBOV infection validated the phenotypic selection in our screens, suggesting that the EBOVΔVP30-EGFP based screen was capable of identifying genes required for EBOV infection. Moreover, our screens unveiled many other potential pro-viral host factors with substantial enrichment, paving the way for further elucidation of their roles in EBOV infection.

### PIK3C3 and SLC39A9 are validated as critical host factors for EBOV infection

**Fig 2:**
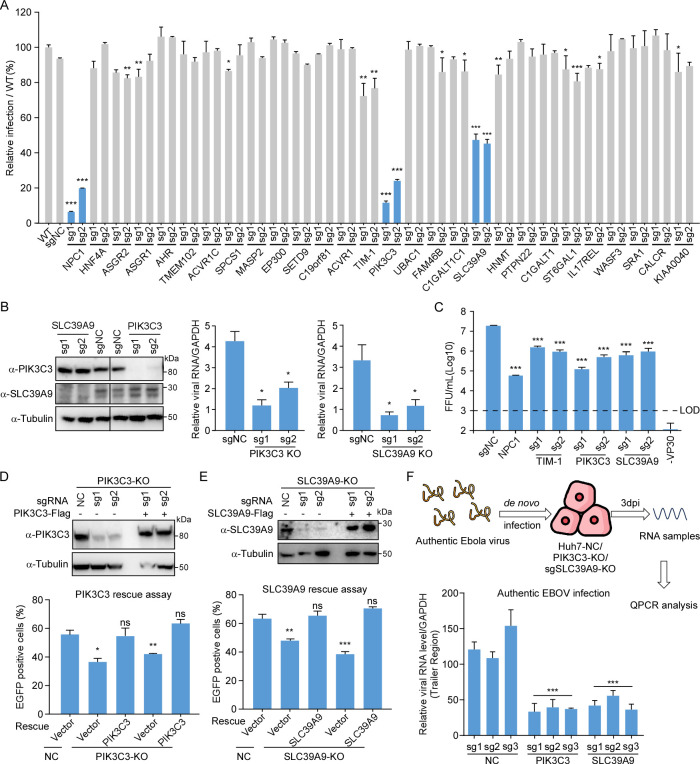
Validation of screen hits. **(A)** Flow cytometry quantification of viral infection in WT and KO Huh7.5.1-VP30 cells. Top-enriched candidate genes, identified through statistical analysis (**S1 Table**), were selected. Huh7.5.1-VP30 cells were transduced with two individual sgRNAs targeting each hit, followed by EBOVΔVP30-EGFP infection at MOI = 1. At 48hpi, cells were fixed, and viral infection (GFP-positive rate) was analyzed by flow cytometry, normalized to infected WT Huh7.5.1-VP30 cells. (**B**) Validation of SLC39A9 and PIK3C3 KO efficiency through immunoblotting (**left**); Huh7.5.1-VP30 cells transduced with sgRNAs were infected with EBOVΔVP30-EGFP virus at MOI = 1. Huh7 WT or KO infected cells were analyzed by RT-qPCR to determine EBOV NP RNA abundance (**right**). **(C)** At 48hpi, supernatants were collected for titration, and focus-forming units per mL (FFU/mL) were calculated. Complementation of Huh7.5.1-VP30 KO cells with respective cDNAs of PIK3C3 (**D**) or SLC39A9 (**E**). SLC39A9 and PIK3C3 protein levels were detected by WB (**upper**), and virus-infected cells were quantified by flow cytometry at 2dpi (**lower**). (**F**) NTC or KO Huh7 cells were challenged with authentic EBOV (MOI = 0.1) for 3 days. RT-qPCR assay determined EBOV RNA abundance, normalized to GAPDH. Error bars represent SD of the mean from one representative experiment with triplicates. Significance assessed by one-way ANOVA, with asterisks indicating significant differences: NS, no significance (p ≥ 0.05); *, p < 0.05; **, p < 0.01; ***, p < 0.001. The experiments were repeated in three independent trials with similar results.

To validate our screen results and identify novel critical host factors, we generated Huh7.5.1-VP30 KO cell lines using two individual sgRNAs targeting each candidate gene. A total of 26 genes were chosen based on their top-ranking in our screens, and viral receptor NPC1 and entry factor TIM-1 were also included as the positive control [18]. The Huh7.5.1-VP30 WT cells or KO cells were infected with EBOVΔVP30-EGFP at MOI = 1, and EGFP expression, serving as a proxy for viral infection, was quantified by flow cytometry analysis at 48 hours post infection (hpi). Upon EBOVΔVP30-EGFP infection, NPC1 KO cells and TIM1 KO cells exhibited significant reductions (80% and 25%, respectively) in viral infection (measured by EGFP percentage), as anticipated. Furthermore, genetic depletion of several other factors, such as ASGR1, ASGR2, and ST6GAL1, also impaired viral infection, albeit with a 10–20% reduction. Notably, the most significant decreases in viral infection were observed with the loss of PIK3C3 and SLC39A9, resulting in a 70% and 50% reduction in viral infection respectively (**Fig 2A**). Moreover, genetic depletion of PIK3C3 or SLC39A9 not only decreased virus infection **(Figs 2A and S2A)** but also significantly diminished EBOV RNA levels in infected cells **(Fig 2B)** and progeny virus production **(Fig 2C)**. Importantly, the genetic complementation of PIK3C3 and SLC39A9 KO cells with their respective cDNAs restored EBOVΔVP30-EGFP viral infection to a level comparable to that of sgNC-transduced cells (**Fig 2D and 2E**), excluding the off-target effect of the sgRNA.

To ascertain that the critical roles of PIK3C3 and SLC39A9 were not specific to the engineered EBOVΔVP30-EGFP virus, we assessed their function in authentic EBOV infection under BSL-4 laboratory conditions. For this purpose, Huh7 NC, PIK3C3 or SLC39A9 KO cells were infected with EBOV (Zaire *Mayinga* strain) at MOI = 0.1. Cells were sampled for RNA extraction, and EBOV RNA abundance was determined by RT-qPCR at 3 days post infection (dpi) (**Fig 2F, upper panel**). Consistent with our results in the EBOVΔVP30-EGFP model (**Fig 2A and 2C**), genetic depletion of PIK3C3 or SLC39A9 efficiently suppressed authentic EBOV infection, as indicated by the decreased EBOV RNA level (**Fig 2F, lower panel**). These findings affirm that PIK3C3 and SLC39A9 are critical host factors in the EBOV life cycle, prompting further investigation into their roles in subsequent studies.

Furthermore, we explored whether PIK3C3 and SLC39A9 functions extend beyond Ebola virus to other viruses. Knockout of PIK3C3 reduced VSV infection, consistent with previous findings that PIK3C3 specific inhibitor could decrease VSV infection [44], emphasizing the importance of endosome trafficking factors like Rab5 and Rab7, essential for both VSV and EBOV infections [45,46]. However, SLC39A9 knockout had no effects on VSV infection. Neither PIK3C3 nor SLC39A9 was necessary for HSV infection (**S2B Fig**). These results suggest that PIK3C3’s important role in endosome trafficking may contribute to various virus infections, despite the distinct entry pathway employed by VSV and EBOV [46]. Conversely, SLC39A9 appears to be a more specific host factor for EBOV infection, although further studies involving a broader range of viruses are warranted for conclusive evidence.

### PIK3C3 and SLC39A9 are implicated in EBOV cell entry

**Fig 3:**
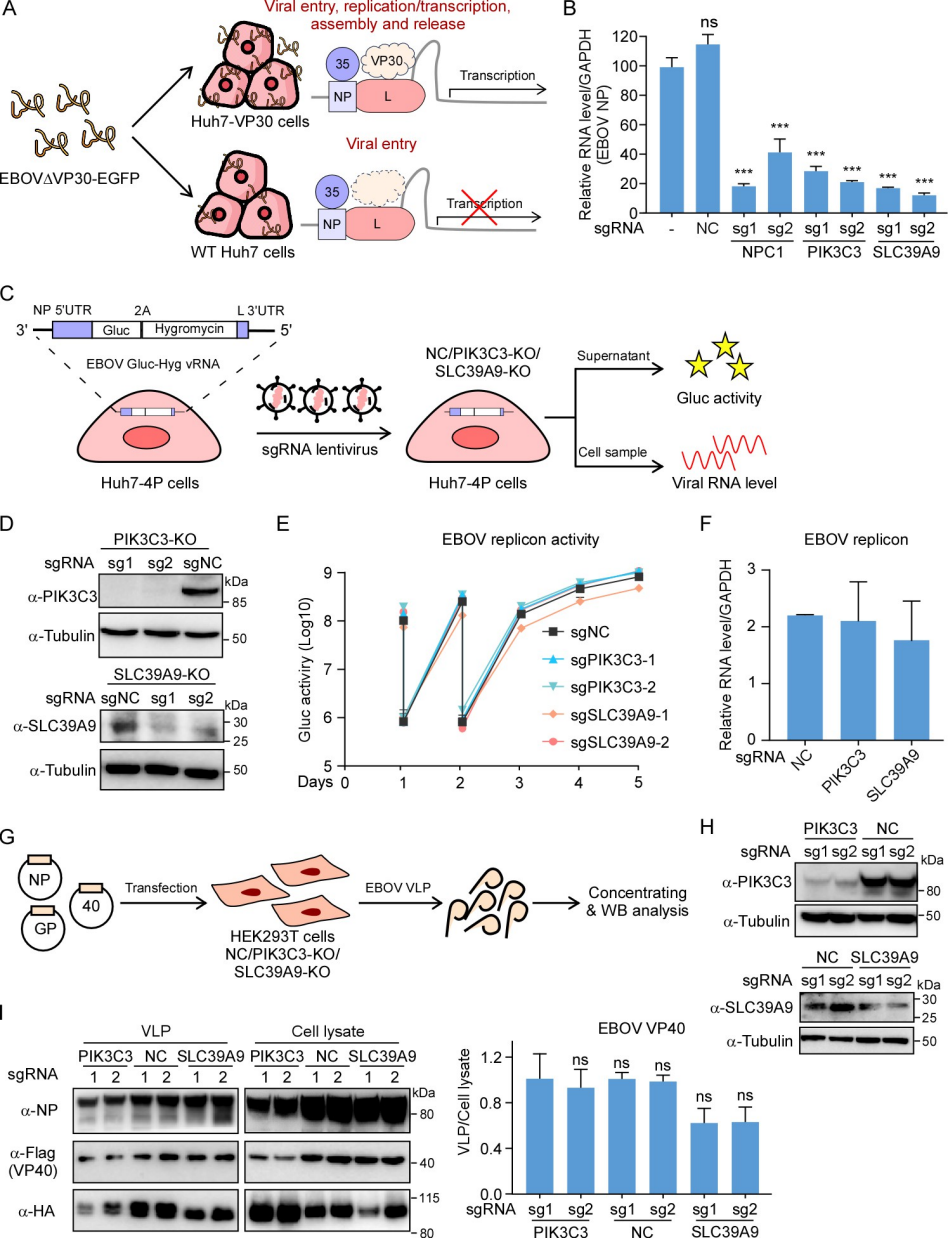
Functional dissection of the roles of SLC39A9 and PIK3C3 in different stages of EBOV infection. (**A**) Huh7 WT or KO cells were inoculated with EBOVΔVP30-EGFP virus at MOI = 10 at 37°C for 3h; absence of transcription factor VP30 in Huh7 cells stuck the virus in the entry step (**lower**). Unbound viruses were washed three times with PBS and cellular RNA was extracted for RT-qPCR analysis. Quantification results are showing on the (**B**). **(C)** Schematic view of the EBOV replicon assay. Huh7-4P replicon cells, transduced with NTC, PIK3C3, or SLC39A9 targeting sgRNAs, were assessed for viral replication through supernatant Gluc activity or cellular viral RNA level. (**D**) KO efficiency of replicon cells detected by PIK3C3 and SLC39A9 immunoblotting. NTC or KO replicon cells were analyzed for Gluc activity **(E)** and RNA level **(F)** after 3 days. (**G**) Schematic view of the EBOV virus-like particle (VLP) production assay. NTC, PIK3C3, or SLC39A9 targeting sgRNAs were transduced into HEK293T cells, and KO efficiency was confirmed by WB (**H**). Co-transfection of EBOV VP40, NP, and GP expression vectors in NTC or KO 293T cells produced VLPs. Concentrated VLP supernatants were collected for WB analysis. (**I**) WB analysis and quantification of concentrated EBOV VLPs. All three viral proteins were detected, and intensity quantification was performed using ImageJ. Experiments were repeated at least three times with similar results; error bars represent the SD of the mean from one representative experiment with triplicates. Significance assessed by one-way ANOVA, with asterisks indicating significant differences: ns, no significance (p ≥ 0.05); *, p < 0.05; **, p < 0.01; ***, p < 0.001.

To understand the mechanisms of PIK3C3 and SLC39A9 regulating EBOV infection, we initially investigated the specific stage of the viral life cycle at which they are involved. To assess whether PIK3C3 and SLC39A9 were implicated in EBOV cell entry, we conducted a viral entry assay with EBOVΔVP30-EGFP virus in which the transcription factor VP30 is replaced with EGFP [35,47]. Since naïve Huh7 cells lack VP30 (WT cells), the entry of EBOVΔVP30-EGFP virus into these cells does not initiate further transcription and replication processes. Consequently, EBOV entry can be evaluated by assessing intracellular viral RNA levels **(Fig 3A, lower**). In detail, we incubated naïve Huh7 cells or Huh7 cells transduced with non-targeting sgRNA (Huh7 sgNC) or specific sgRNAs targeting NPC1, PIK3C3, or SLC39A9 with EBOVΔVP30-EGFP virus (MOI = 10) at 37°C for 3h to allow virus entry into cells. This was followed by extensive washing to remove the unbound virus and subsequent purification of intracellular RNA. Results showed that the loss of NPC1 significantly decreased cellular viral RNA level, as expected. Furthermore, the loss of PIK3C3 or SLC39A9 also significantly reduced viral entry, represented by a much lower level of EBOV RNA compared to WT or sgNC cells (**Fig 3B**). Meanwhile, genetic depletion of PIK3C3 or SLC39A9 did not impact NPC1’s mRNA or protein levels (**S3A and S3B Fig**). These data suggest that PIK3C3 and SLC39A9 are both essential for efficient EBOV virus entry.

Next, we assessed the roles of PIK3C3 and SLC39A9 in EBOV replication and transcription. To this end, we electroporated the *in vitro* transcribed minigenome replicon vRNA harboring Gaussia luciferase and hygromycin-resistant reporter genes into Huh7-4P cells (Huh7 cells expressing viral NP, VP30, VP35, L genes) (**Figs 3C and S4A**). The cells were then cultured with hygromycin to establish replicon cells [48]. The replicon RNA level, indicative of active EBOV replication, was detectable and suppressed by remdesivir in a dose-dependent manner (**S4B Fig**). Subsequently, these replicon cells were transduced with PIK3C3 and SLC39A9 specific sgRNAs or non-targeting sgRNAs. Supernatant Gluc activity and cellular replicon RNA levels were determined to reflect viral replication levels (**Fig 3C and 3D**). Notably, the knockout of PIK3C3 or SLC39A9 did not impact replicon Gluc activity or replicon RNA levels (**Fig 3E and 3F**), providing evidence that PIK3C3 or SLC39A9 is not essential for viral replication and transcription.

It has been reported that EBOV virus-like particles (VLPs) can be generated by co-expressing viral nucleoprotein (NP), matrix protein (VP40), and glycoprotein (GP) [49–51]. Therefore, we utilized a VLP production assay to evaluate the role of PIK3C3 and SLC39A9 in EBOV virus assembly and budding. HEK293T cells transduced with non-targeting or gene-specific sgRNAs (PIK3C3 or SLC39A9) were transfected with EBOV VP40, NP and GP, and the resulting VLPs were concentrated for Western blot analysis (**Fig 3G**). Results demonstrated that the KO of PIK3C3 or SLC39A9 did not significantly decrease VLP production (**Figs 3H, 3I and S4C)**. Collectively, these findings indicate that PIK3C3 and SLC39A9 are implicated in viral entry but not in other stages of the EBOV life cycle.

### PIK3C3’s kinase activity is required for EBOV entry

**Fig 4:**
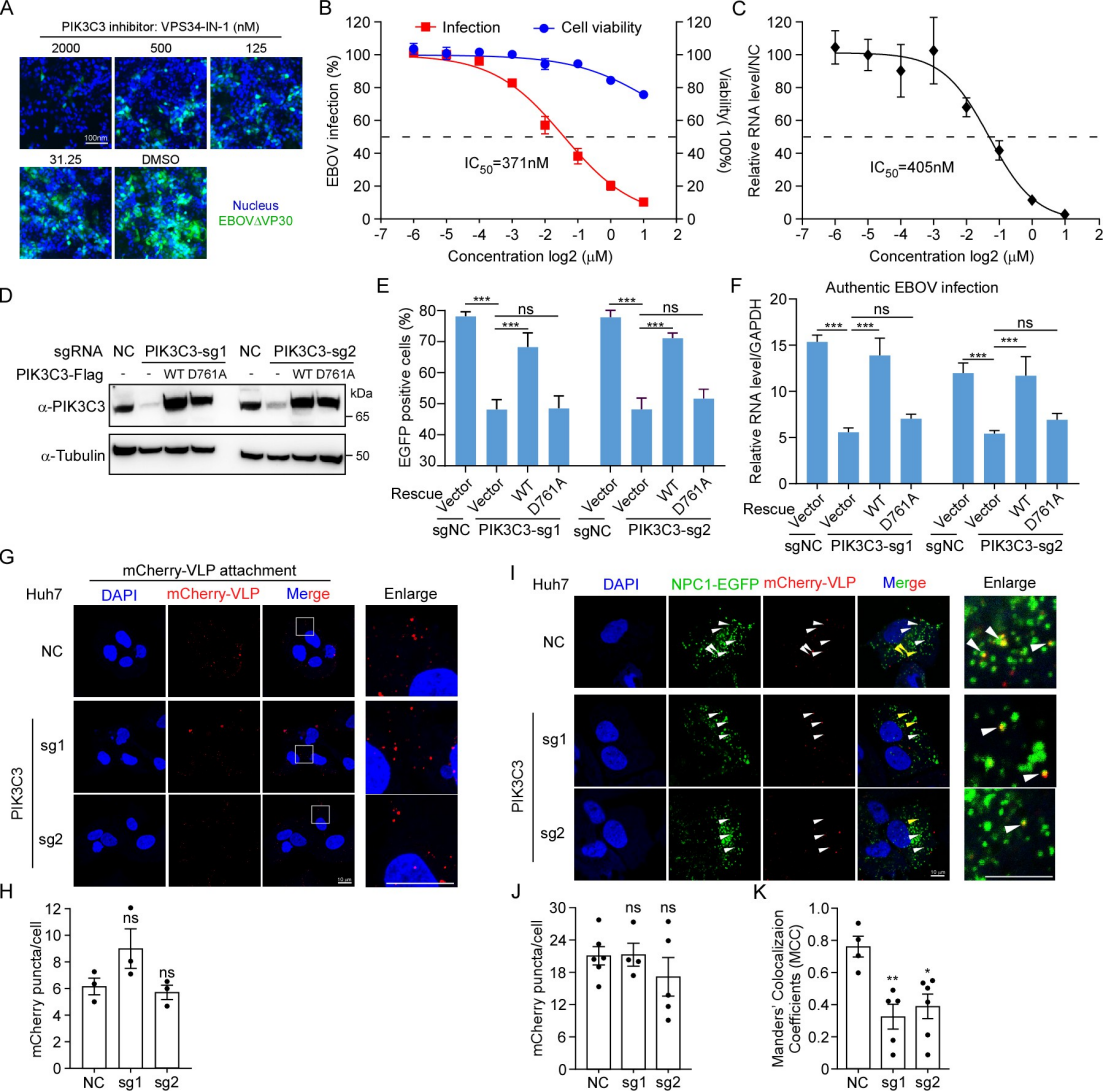
Kinase activity of PIK3C3 was required for trafficking of EBOV virions to NPC1 containing compartments. **(A)** Huh7.5.1-VP30 cells were infected with EBOVΔVP30-EGFP (MOI = 1) in the presence of PIK3C3 inhibitor VPS34-IN-1 for 2 days. **(B)** EGFP was quantified by flow cytometry to determine the inhibitory effect. Cell viability was determined without infection using the CellTiter-Glo Luminescent Cell Viability kit (Promega). **(C)** Huh7.5.1-VP30 cells were challenged by authentic EBOV (MOI = 0.1) for 3 days. Viral RNA level was quantified by RT-qPCR assay. A sigmoidal dose–response curve was fitted to the data using Prism GraphPad 8.0 (GraphPad Software). The IC50 value was calculated from the sigmoidal function. **(D)** Huh7.5.1-VP30 cells with PIK3C3 knocked out were transduced with WT or D761A mutated PIK3C3. Protein expression was confirmed by Western blotting. **(E)** Following transduction, cells were infected with EBOVΔVP30-EGFP (MOI = 1) for 2 days, and EGFP expression was quantified using flow cytometry. **(F)** Transduced cells were infected with authentic EBOV(MOI = 0.1) for 3 days, and the viral RNA level was measured by RT-qPCR assay. **(G)** Huh7 WT or KO cells was incubated with 20 μL mCherry tagged VLP (F535R) for 1h at 4°C. Unbound VLPs was then washed and cells were fixed and stained with DAPI for Confocal analysis**. (H)** Quantification of mCherry-VLP puncta in **(G)** by ImageJ. Each point represents one image field. **(I)** Huh7-NPC1-EGFP WT or KO cells was incubated with 20 μL mCherry tagged VLP (F535R) for 3h at 37°C. Unbound VLPs was then washed and cells were fixed and stained with DAPI for confocal analysis. (Scale bar: 10 μm.) **(J)** Quantification of mCherry-VLP puncta in **(I)** by ImageJ. **(K)** Manders’ Colocalization Coefficiency (MCC) of NPC1-EGFP and mCherry-VLP, which represents ratio of colocalization area to total mCherry-VLP area. Calculated by ImageJ ‐ “Coloc2” plugin. Four to six captured image fields were quantified for each group. Error bars represent mean ± SEM. Significance assessed by one-way ANOVA, the asterisks represent significant differences: ns, no significance (p ≥ 0.05); *, p < 0.05; **, p< 0.01; ***, p< 0.001.

PIK3C3, also named as VPS34 is a Class III phosphoinositide 3-kinase (PI3K) capable of phosphorylating phosphatidylinositols (PtdIns) to generate phosphatidylinositol 3-phosphate (PtdIns(*3*)P), thereby regulating endocytic and autophagic membrane traffic [52]. This enzyme has demonstrated functionality in various RNA viruses, including hepatitis C virus (HCV) [53], tombusvirus (TBSV) [54], and SARS-CoV-2 infection [55]. To investigate the indispensability of PIK3C3’s kinase activity in EBOV infection, we employed a selective PIK3C3 kinase inhibitor, VPS34-IN-1 [53], and assessed its efficacy against EBOVΔVP30-EGFP infection. To this end, Huh7-VP30 cells were infected with EBOVΔVP30-EGFP (MOI = 1) in the presence of varying doses of VPS34-IN-1. After a 2-day infection period, cells were harvested for flow cytometry analysis to quantify EGFP expression as an indicator of viral infection. Our results demonstrated that VPS34-IN-1 effectively impeded EBOVΔVP30-EGFP infection in a dose-dependent manner (IC_50_ = 371nM) with negligible cellular toxicity (**Fig 4A and 4B**). Furthermore, the antiviral efficacy of VPS34-IN-1 was further confirmed under authentic EBOV infection, exhibiting a comparable IC_50_ = 405nM (**Fig 4C**). Moreover, to demonstrate the necessity of PIK3C3 kinase activity in EBOV infection, we generated a kinase-inactive form of PIK3C3 harboring a D761A point mutation in its activation loop (**Fig 4D**) [56,57]. Unlike WT PIK3C3, complementation of PIK3C3 KO cells with PIK3C3-D761A failed to rescue the reduction in EBOV infection caused by PIK3C3 KO (**Fig 4E**). This result was further confirmed under authentic EBOV infection, wherein only WT PIK3C3 could restore viral RNA levels (**Fig 4F**), emphasizing the critical role of PIK3C3 kinase activity in EBOV infection.

As PIK3C3’s function is implicated in endocytic and autophagic membrane trafficking, and it has been reported that autophagy pathway is essential for EBOV internalization following virion attached to the cell plasma membrane [47], which is line with our results that PIK3C3 could promote EBOV cell entry (**Fig 3A and 3B**). To further elaborate the function process of PIK3C3 in EBOV entry, we take advantage of an fusion deficient GP mutant (F535R) [58,59]. EBOV VLP bearing GP-F535R could undergo macropinocytosis, trafficking to NPC1 containing endolysosomes, cleavage by cathepsin B and L, binding to NPC1 but without further membrane fusion [60]. Thus, we packaged mCherry-tagged VLPs with mCherry-tagged VP40, NP and GP-F535R to analyze EBOV entry steps before membrane fusion. We first evaluate whether PIK3C3 was required for virion attachment. Huh7 NC or PIK3C3 KO cells were incubated with fusion-deficient mCherry-VLP (F535R) at 4°C for 1h (to prevent viral entry and fusion). Unbound VLPs were washed and cells were fixed for confocal analysis. Results showed that the amounts of mCherry-VLP puncta binding to NC or KO cells were comparable indicating that PIK3C3 was not required in EBOV attachment (**Fig 4G and 4H**). Next, we evaluate the virus internalization and trafficking process with prolonged incubation (37°C, 3h) of fusion-deficient VLPs with NC or PIK3C3 KO Huh7-NPC1-EGFP cells. Quantification of mCherry puncta showed that entered VLP level was not obviously affected by PIK3C3 loss (**Fig 4I and 4J**). However, we found that the ratio of VLPs colocalizing with NPC1 was significantly lower in those PIK3C3 KO cells compared to NC cells (**Fig 4I and 4K**). Collectively, these findings suggest that EBOV exploits or reprograms endocytic and autophagic membrane trafficking, regulated by PIK3C3, for cell entry subsequent to virion binding to the cell surface. Consequently, PIK3C3 emerges as a promising drug target for inhibiting EBOV infection.

### SLC39A9 is capable of binding to the viral glycoprotein and facilitating virion attachment

**Fig 5:**
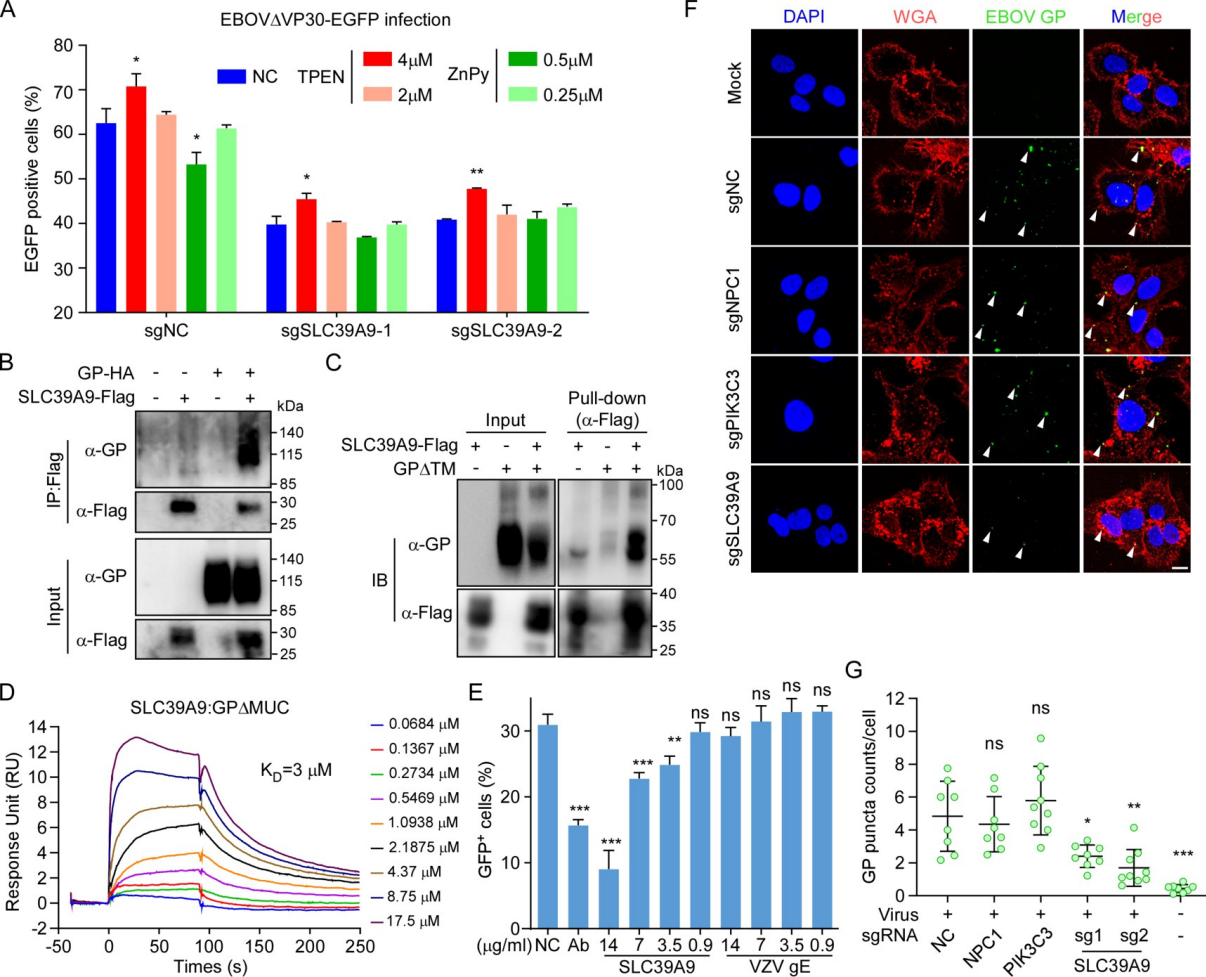
SLC39A9 could bind to EBOV GP mediating viral attachment. **(A)** WT or KO Huh7.5.1-VP30 cells were infected with EBOVΔVP30-EGFP (MOI = 1) in the presence of TPEN or ZnPy for 2 days. EGFP was quantified by flow cytometry to determine the inhibitory effect. **(B)** HEK293T cells were co-transfected with SLC39A9-Flag and EBOV GP-HA. 2 days post transfection, cell lysates were immunoprecipitated with anti-Flag M2 magnetic beads and immunoblotted with indicated antibodies. **(C)** Pull-down assay. 10 μg purified SLC39A9-Flag and GPΔMUC-His proteins were incubated at 4°C for 4h together with Flag M2 beads before immunoblotting. **(D)** BIAcore diagram of purified GPΔMUC bound to SLC39A9 protein. Ligand: analyte was presented. Ligand proteins were captured on the chip, and serial dilutions of lysate proteins were then injected over the chip surface. The binding affinity K_D_ values were calculated by the BIAcore 3000 analysis software (BIAevaluation Version 4.1). **(E)** EBOVΔVP30-EGFP was pre-incubated with mock or indicated doses of SLC39A9 or VZV gE protein or 14 μg/ml EBOV neutralizing Ab Q206 at 37°C for 1h. Huh7.5.1-VP30 cells were then inoculated with a final MOI = 0.1. Two days post infection, EGFP was quantified with Flow cytometry. **(F)** Huh7 WT or KO cells were inoculated with EBOVΔVP30-EGFP virus and incubated at 4°C for 1h. Cells were then washed, fixed and stained with EBOV GP Ab, WGA (cell membrane) and DAPI (nucleus). Sections along the z-axis were collected with LSM880 (Carl Zeiss). Figures were representative results by maximum projection. Scale bars, 10 μm. **(G)** Quantification of views from **(E)** by ImageJ. Each point represents result from one captured image field. Error bars represent mean ± SEM. Significance assessed by one-way ANOVA, the asterisks represent significant differences: ns, no significance (p ≥ 0.05); *, p < 0.05; **, p< 0.01; ***, p< 0.001.

SLC39A9 functions as a membrane androgen receptor (mAR) coupled to G proteins, and is a member of the SLC39 family (SLC39A9, solute carrier family 39 member 9; also known as Zrt- and Irt-like protein 9, ZIP9), acting as a zinc transporter protein that facilitates the transport zinc from extracellular environment into cytosol [61]. Despite limited studies on this protein and its rarely described role in viral infection, our initial investigation focused on determining whether the zinc transport function and overall cellular zinc states impact viral infection. Zinc (Zn) binds to Zinc-finger transcription factors, leading to the upregulation of metallothionein genes, such as MT1A and MT2A [62–64]. Consequently, MT1A and MT2A mRNA levels serve as biological indicators of cellular zinc levels. We successfully modulated overall cellular zinc levels by either decreasing or increasing them through the supplementation of the cell culture medium with a zinc ion chelator (TPEN) or a zinc ionophore (ZnPy), respectively (**S5A and S5B Fig**). While changes in cellular zinc levels slightly affected EBOV infection in both WT and SLC39A9 KO cells, neither the increase nor decrease in cellular zinc levels could rescue the decreased viral infection caused by SLC39A9 depletion (**Fig 5A**). These findings suggest that SLC39A9 is unlikely to regulate EBOV infection through alterations in cellular zinc levels, a conclusion further supported by the absence of enrichment of other zinc transporter proteins in our screens (**S1 Table**).

SLC39A9, identified as a membrane protein predominantly located in the plasma membrane, endoplasmic reticulum, and mitochondrial membrane structures [61], was confirmed to localize in the plasma membrane of Huh7 cells (**S6A Fig**). This led us to propose that SLC39A9 might directly interact with EBOV GP to facilitate viral entry. To investigate this possibility, we initially explored the association between SLC39A9 and EBOV GP through a co-immunoprecipitation (co-IP) assay. HEK293T cells were transfected with SLC39A9-Flag and/or EBOV GP-HA cDNA, and cell lysates were collected for the co-IP assay 2 days post-transfection. As shown in **Fig 5B**, SLC39A9-Flag co-precipitated with EBOV GP-HA protein, confirming their interaction. Subsequently, we conducted an *in vitro* pull-down assay to further examine the direct binding capability of SLC39A9 to GP. Recombinant SLC39A9-Flag and GPΔMUC-His (GP lack of transmembrane and mucin domains) proteins were purified from 293FT cells (**S6B and S6F Fig**), mixed or kept separate *in vitro*, and subjected to incubation. Immunoblotting analysis demonstrated that GPΔMUC-His was pulled down by Flag M2 antibodies in the presence of SLC39A9-Flag, confirming a direct interaction between SLC39A9 and EBOV GP protein (**Fig 5C**). In addition, we explored the binding affinity between these two proteins by surface plasmon resonance (SPR) assays. Results revealed that EBOV GP could bind to SLC39A9 with a moderate binding affinity of 3 μM (**Fig 5D**). Furthermore, our findings revealed that recombinant SLC39A9 could inhibit EBOVΔVP30-EGFP infection in a dose-dependent manner, whereas an unrelated Varicella-Zoster Virus (VZV) glycoprotein E (VZV gE) showed no such effect (**Fig 5E**). This suggests that recombinant SLC39A9 may compete with plasma membrane-localized SLC39A9 for interaction with EBOV, thereby blocking viral infection.

Subsequently, to determine if SLC39A9 facilitates virus attachment to the cell plasma membrane by interacting with viral GP, we incubated Huh7 NC, SLC39A9 KO, PIK3C3 KO or NPC1 KO cells with EBOVΔVP30-EGFP virus at 4°C for 1 hour. After extensive washing to remove unbound viruses, cells were stained for cell surface-bound viral particles using GP antibody Q206 [65–67]. No difference was observed between PIK3C3 KO, NPC1 KO and NTC cells, suggesting that PIK3C3 and NPC1 are not required for viral particle attachment, consistent with previous VLP attachment assay (**Fig 3C and 3D**). In contrast, Huh7 SLC39A9 KO cells exhibited a significant decrease in GP staining compared to NC cells, indicating that SLC39A9 is essential for EBOV virus attachment in the early entry step (**Fig 5F and 5G**). Collectively, these findings reveal that SLC39A9 serves as an attachment factor during the early entry phase and facilitates viral entry through direct interaction with EBOV GP.

### SLC39A9 showed direct interaction with NPC1 C-terminal domain

**Fig 6:**
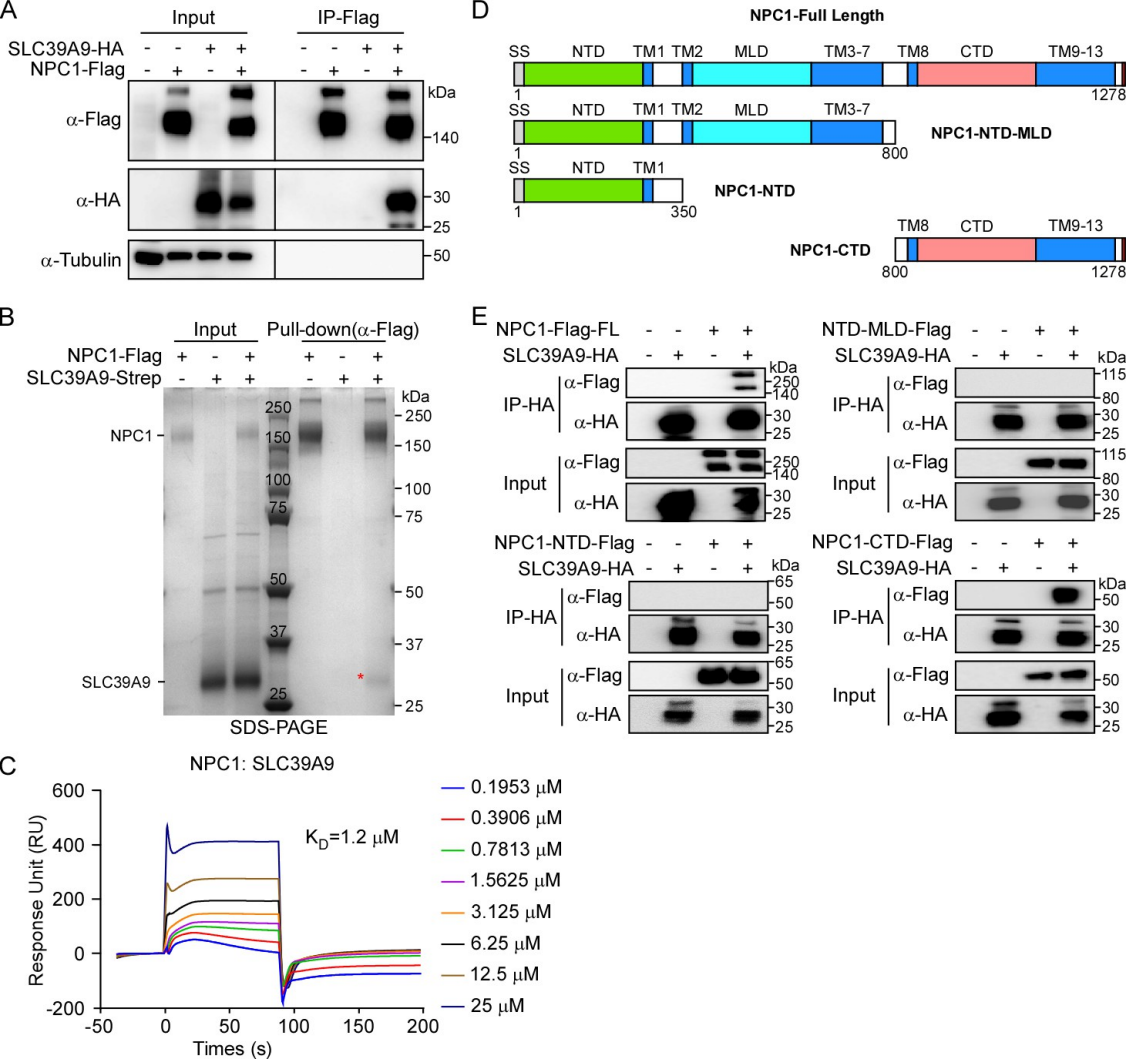
Interaction dissection between NPC1 and SLC39A9. **(A)** HEK293T cells were co-transfected with NPC1-Flag and EBOV SLC39A9-HA. 2 days post transfection, cell lysates were immunoprecipitated with anti-Flag M2 magnetic beads and immunoblotted with indicated antibodies. **(B)** SDS-PAGE results of pull-down assay between NPC1 and SLC39A9: 10 μg purified NPC1-Flag and SLC39A9-Strep proteins were incubated at 4°C for 4h together with Flag M2 beads. **(C)** BIAcore diagram of purified SLC39A9 bound to NPC1 protein. Ligand: analyte was presented. **(D)** Schematic representation of NPC1 and its truncations. Each construct contains a C-terminal Flag tag. **(E)** Interaction detection between NPC1-Flag constructs and SLC39A9-HA by Co-IP assay. Co-transfected (2 days) HEK293T cell lysates were subjected to immunoprecipitation with anti-HA-Nanoab-Magnetic beads. This experiment was independently repeated three times with similar results.

During the validation of SLC39A9’s cellular localization [61], we observed that SLC39A9 exhibited apparent colocalization with viral receptor NPC1 protein, especially in lysosome and Golgi compartments **(S7A Fig)**. To further explore their association, we conducted co-IP and *in vitro* pull-down assays. HEK293T cells were transfected with SLC39A9-HA and/or NPC1-Flag cDNA and cell lysates were collected for the co-IP assay 2 days post-transfection. Our results indicated that NPC1-Flag co-precipitated with SLC39A9-HA protein, confirming their interaction (**Fig 6A**). Next, we purified recombinant SLC39A9-Strep and NPC1-Flag proteins (**S7B Fig**) and these proteins were then either mixed or kept separate *in vitro*. SDS-PAGE analysis revealed that SLC39A9-Strep was pulled down by Flag M2 antibodies in the presence of NPC1-Flag (**Fig 6B**). Furthermore, we performed the SPR assay to measure the binding affinity between the NPC1 and SLC39A9, which was about 1.2 μM (**Fig 6C**). These experiments provided evidence for a direct interaction between SLC39A9 and NPC1.

To further dissect the interaction domain of NPC1 responsible for binding with SLC39A9, we generated three NPC1-Flag truncations (NPC1-NTD-MLD: 1-800aa, NPC1-NTD: 1-350aa, and NPC1-CTD: 800-1278aa) (**Fig 6D**) and co-transfected WT or each truncation with SLC39A9-HA cDNAs into HEK293T cells. Subsequent co-IP with SLC39A9-HA revealed that only WT NPC1 and truncation containing NPC1-CTD could interact with SLC39A9-HA, indicating that the C-terminal domains (800-1278aa) of NPC1 are necessary and sufficient for SLC39A9 interaction (**Fig 6E**). As the NPC1 MLD domain was responsible for EBOV GPcl binding which was the 19kD cleaved form of GP catalyzed by CSTB and CSTL [68,69], this data suggests that SLC39A9 might act as a mediator between NPC1 and GP, rather than a binding competitor.

### SLC39A9 facilitates the interaction between the viral glycoprotein and its cellular receptor NPC1

**Fig 7:**
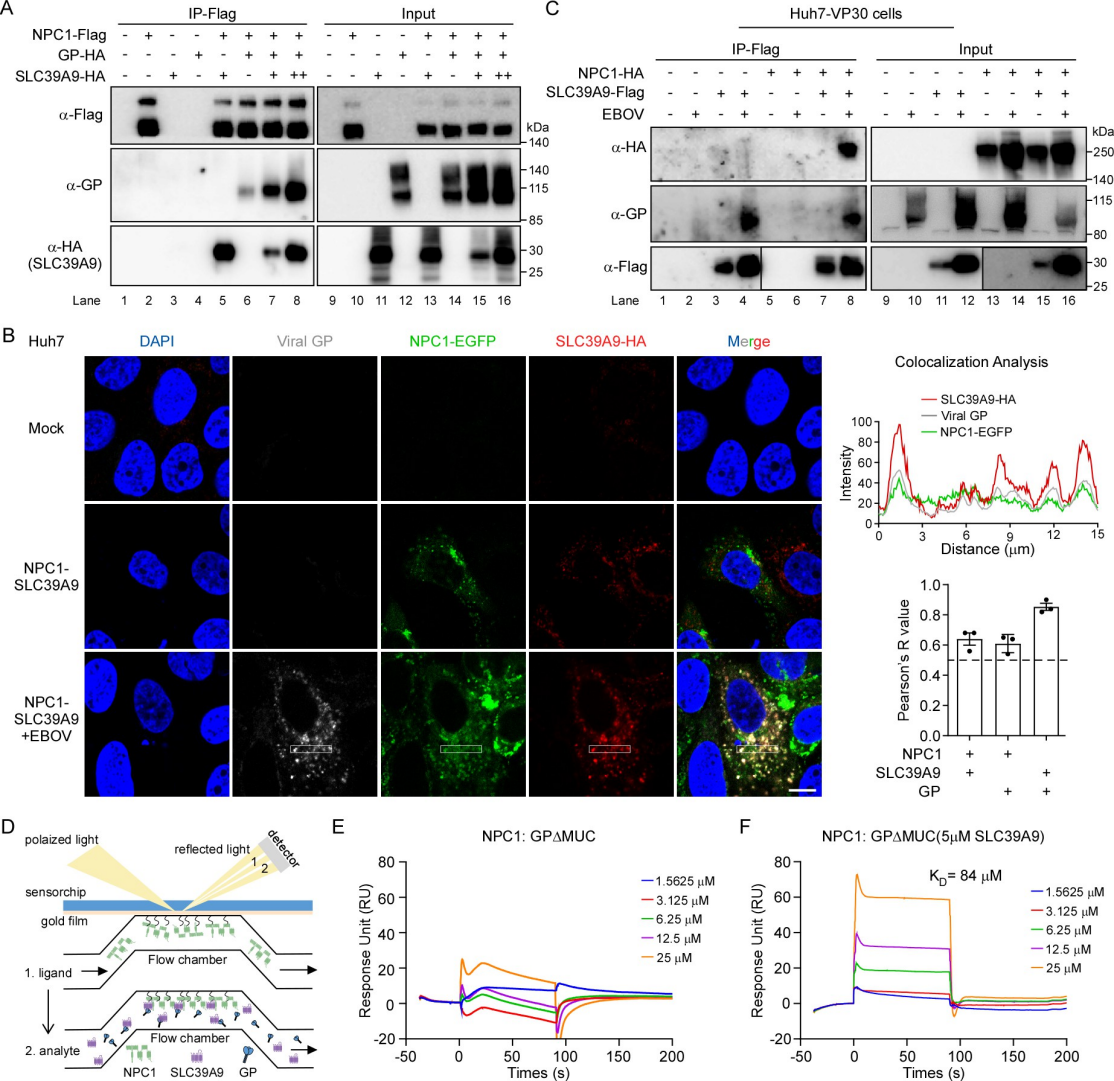
SLC39A9 could promote interaction between EBOV GP and its host receptor NPC1. **(A)** NPC1-Flag, GP-HA were co-transfected with an increase dose of SLC39A9-HA into 293T cells for 2 days. Cell lysates were then subjected for immunoprecipitation assay with Flag-M2 magnetic beads and detected with indicated Abs. Scale bars, 10 μm. **(B)** Huh7 cells stably expressing NPC1-EGFP and SLC39A9-HA proteins were infected with EBOVΔVP30-EGFP virus (3h at 37°C) and cells were fixed and stained with EBOV GP Ab and SLC39A9-HA. Confocal images were collected with LSM880 (Carl Zeiss) at 63× lens. Co-localization analysis was processed with ImageJ- “Plot Profiles” and “Coloc2” (**right**). **(C)** Huh7-VP30 cells stably transduced with NPC1-EGFP-HA and SLC39A9-Flag was infected with EBOVΔVP30-EGFP virus (MOI = 1, 2 days). Cell lysates were subjected for immunoprecipitation analysis with Flag-M2 magnetic beads. Proteins were detected with indicated Abs. **(D)** Schematic diagram of SPR assay, NPC1 was first immobilized onto the chip followed by influx of GP alone or together with SLC39A9**. (E)** BIAcore diagram of purified GPΔMUC bound to the full-length NPC1 protein. **(F)** BIAcore diagram of purified GPΔMUC bound to the full-length NPC1 protein with the presence of 5 μM SLC39A9 protein. NPC1 proteins were captured on the chip, and serial dilutions of GPΔMUC were then injected over the chip surface. The binding affinity K_D_ values were calculated by the BIAcore 3000 analysis software (BIAevaluation Version 4.1). This experiment was independently repeated twice with similar results.

Intrigued by the observation that SLC39A9 interacts with both NPC1 and EBOV GP, we speculated whether SLC39A9 also plays a role in the late entry step, in addition to its function in promoting virion attachment (**Fig 5E–5G**). To test this possibility, we initially performed a co-IP assay to test the potential association of EBOV GP, SLC39A9, and NPC1. HEK293T cells were transfected with NPC1-Flag, GP-HA or SLC39A9-HA in various combinations, and after 2 days, cell lysates were collected for the co-IP assay by immunoprecipitation of NPC1-Flag. As expected, NPC1-Flag co-immunoprecipitated with SLC39A9-HA (**Fig 7A, lane 5**), but the association with GP-HA was relatively weak (**Fig 7A, lane 6**), possibly mediated by endogenous SLC39A9 in HEK293T cells. Notably, an increased co-precipitation of GP-HA by NPC1-Flag was observed as the expression of SLC39A9-HA increased (**Fig 7A, lane 7–8 vs lane 6**). These findings suggest that SLC39A9 could enhance the interaction between EBOV GP and the cellular receptor NPC1.

To strengthen this point in the context of viral infection, we infected the Huh7-NPC1-EGFP/SLC39A9-Flag cells with EBOVΔVP30-EGFP or not. After 3h, the cells were fixed for immunostaining to probe the localization of viral particles (viral GP), NPC1-EGFP and SLC39A9-HA, respectively. Our data showed that SLC39A9, NPC1 and viral GP could co-localized upon viral infection (**Fig 7B**). Moreover, we transduced the Huh7-VP30 cells with NPC1-HA and SLC39A9-Flag cDNAs, and then the cells were inoculated with EBOVΔVP30-EGFP virus. After 2 days, the cell lysates were collected for immunoprecipitation of SLC39A9-Flag. Our data indicated that NPC1 and SLC39A9 could interact with viral GP separately (**S8A and S8B Fig**). Moreover, SLC39A9-Flag specifically co-precipitated with NPC1-HA and viral GP proteins upon EBOVΔVP30-EGFP infection (**Fig 7C, lane 8 vs. lane 7**), further confirming their interaction in the context of virus infection. These findings collectively demonstrate that SLC39A9 can form a complex with both NPC1 and EBOV viral GP, thereby promoting interaction between NPC1 and unprimed viral GP before being cleaved by CSTB and CSTL.

To assess the active interaction among the three proteins, we conducted SPR assays to explore the role of SLC39A9 in this complex (**Fig 7D**). As reported [68,69], only the primed GPcl cleaved by thermolysin *in vitro*, showed binding affinity (K_D_ = 18.5 μM) to NPC1 (**S9A–S9D Fig**); in contrast, unprimed GPΔMUC did not bind to NPC1 (**Fig 7E**), and SLC39A9 exclusively interact with unprimed GPΔMUC, but not primed GPcl (**S9E Fig**). However, when we maintained a constant concentration of SLC39A9 (5 μM) at different concentrations of GPΔMUC, a dose-dependent response was observed between flow GPΔMUC and immobilized NPC1 with a binding affinity of approximately 84 μM (**Fig 7F**). To further elucidate the functional role of the interaction between SLC39A9 and GP in Ebola virus infection, we treated EBOVΔVP30-EGFP viral particles with thermolysin (THL) to cleave virion surface glycoproteins, generating GPcl-bearing particles. Western blot analysis confirmed successful glycoprotein cleavage, showing ~19kD GPcl on THL-treated particles, while internal NP protein remained unaffected (**S9F Fig**). Subsequent infection of Huh7.5.1-VP30 cells with cleaved or uncleaved viruses, followed by assessment after two days using flow cytometry, demonstrating that SLC39A9 deficiency significantly reduced EBOVΔVP30-EGFP virus infection, consistent with our results. Interestingly, GPcl-cleaved virus exhibited significantly increased infection compared to uncleaved virus (**S9G Fig**). These results emphasize the crucial role of the SLC39A9-GP interaction in facilitating Ebola virus infection, reinforcing our proposed mechanism of SLC39A9 action.

**Fig 8:**
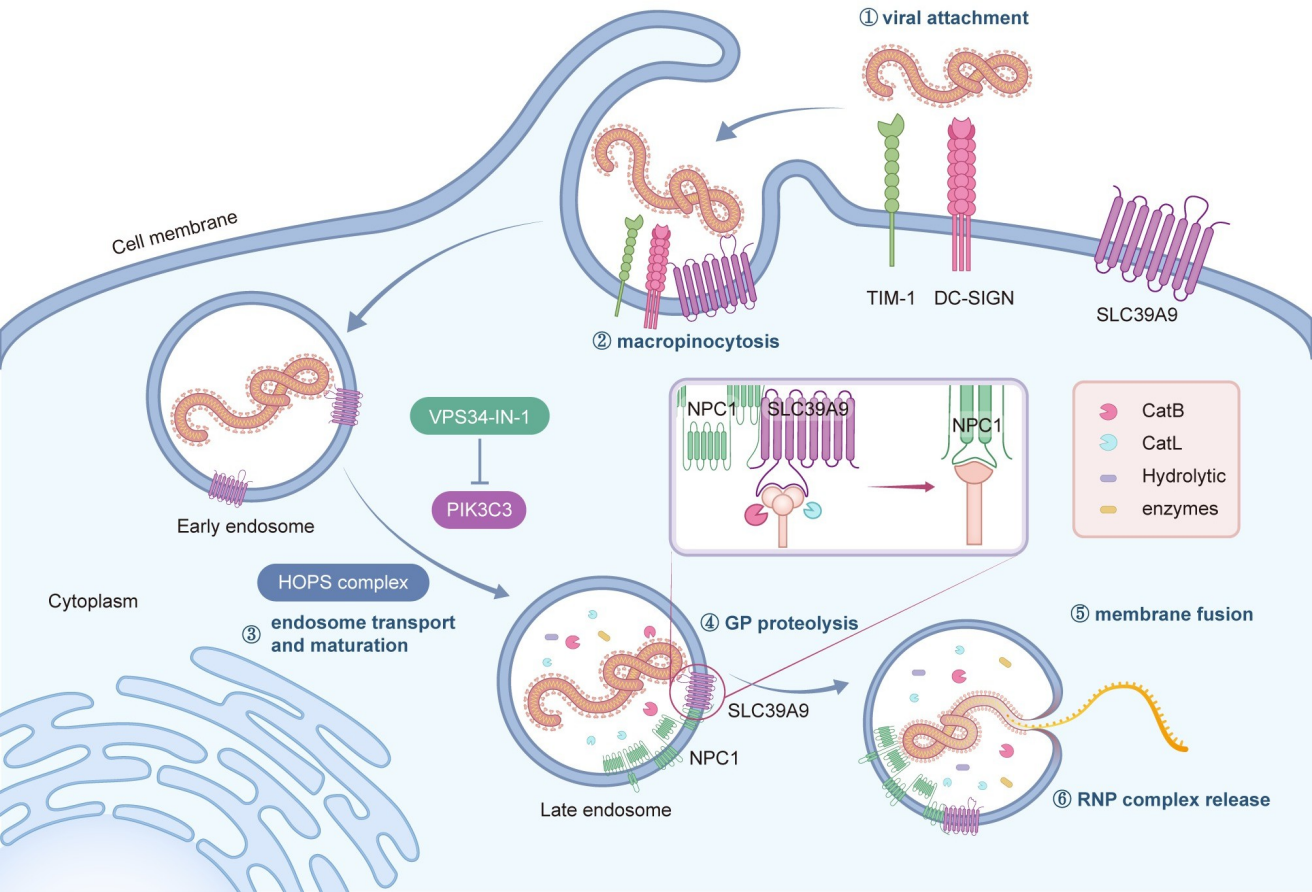
Proposed model of PIK3C3 and SLC39A9 in EBOV entry. Besides those known attachment factors like TIM-1, DC-SIGNs etc, SLC39A9 could facilitate the attachment of Ebola virus on the cell surface. After macropinocytosis, viruses were internalized into endosomes. With the maturation of virus containing endosomes which PIK3C3 seems to participate in besides known HOPS complex, SLC39A9 interacts with NPC1 and uncleaved viral GP providing a favorable distance between NPC1 and GP. Once the GP cap was removed by the Cathepsin B and L, NPC1 could bind to the exposed GP RBD and induce membrane fusion. Finally viral RNA genome together with NP, VP35, VP30, L (ribonucleoprotein (RNP) complex) could be released into cytoplasm and initiate further viral life cycle.

Taken together, our findings indicate that cell plasma membrane-localized SLC39A9 engages in a direct interaction with EBOV GP, facilitating the attachment of viral particles. Subsequently, the SLC39A9-viral particle complexes undergo internalization through macropinocytosis into endosomes, followed by endosome trafficking and maturation as endolysosomes, a process regulated by PIK3C3. The SLC39A9-viral particle complex further interacts with NPC1, bridging the interaction of GP with the cellular receptor NPC1. Further cleavage of viral GP by cathepsins generates primed GP (GPcl), which directly interacts with NPC1, initiating subsequent membrane fusion and RNP release events (**Fig 8**).

## Discussion

Due to the high pathogenicity and broad tropism of EBOV, it is crucial to identify key host factors involved in the viral lifecycle for a comprehensive understanding of viral zoonosis, pathogenesis, and spread. Previous studies have implicated various cellular factors in the EBOV life cycle using diverse approaches. NPC1, identified through a genetic screen, acts as the cellular receptor for EBOV, while TIM1, discovered through comparative genetics analysis, functions as an attachment receptor, facilitating viral entry. In addition, DC-SIGN and Tyro3 protein kinases, discovered by cDNA overexpression, are able to promote viral attachment. Recently, whole genome CRISPR/Cas9 genetic screens have emerged as powerful tools for comprehensive identification of host factors essential for virus life cycles. Many groups have successfully utilized pooled or genome-wide CRISPR/Cas9 screen strategy and identified various host factors in viral infections [70–75]. In this study, we leveraged the BSL-2 accessible EBOV cell culture system to conduct a genome-wide CRISPR/Cas9 screen, leading to the identification of several crucial host factors for EBOV infection.

In addition to the well-established host factors highly enriched in our screen, we identified two novel host factors, PIK3C3 and SLC39A9. Genetic depletion of PIK3C3 or SLC39A9 resulted in reduced EBOV infection, which has been validated in EBOVΔVP30-EGFP and authentic EBOV cell culture models. By using EBOV pseudotyped virus, minireplicon and VLP models which could mimic viral entry, replication and virus assembly and release respectively, we found that PIK3C3 and SLC39A9 function to promote virus cell entry.

EBOV virions enter cells through macropinocytosis [24,45], the earliest reported form of endocytosis. Recent findings have implicated autophagy proteins, such as Beclin-1, autophagy-related protein 7, and LC3B, in macropinocytosis [76], are critical for the internalization of EBOV virus [47]. Our study identifies PIK3C3, a pivotal protein known for its role in endosome trafficking and autophagy [77–81], as an essential factor for EBOV infection. As macropinocytosis involves multiple stages, including macropinosome formation, endosome trafficking, lysosome fusion, and recycling to the cell surface [82], further research is needed to pinpoint which of these steps is the primary target of PIK3C3. The efficient reduction of virus infection with negligible cytotoxicity upon inhibiting PIK3C3 kinase activity using specific inhibitors highlights its potential as a promising host target for antiviral therapy. Additionally, it remains to be elucidated whether PIK3C3 is crucial for other viruses utilizing the same macropinocytosis pathway [25,83,84].

SLC39A9 is an unexplored member of the zinc transporter family and a recently identified novel androgen receptor with reported localization on the plasma membrane, endoplasmic reticulum, and mitochondrial membrane structures [61,85]. Our findings reveal that SLC39A9 also localizes on the lysosome membrane (**S7A Fig)** and functions not only as an attachment factor on the cellular membrane for EBOV virions at the early stage of viral entry but also plays a crucial role at the late stage through direct interactions with both viral GP and cellular receptor NPC1 in the late endolysosome. In detail, the EBOV virion attaches to SLC39A9 at the cell surface, facilitated by the interaction between viral GP and SLC39A9. The virion-SLC39A9 complex is then internalized into the cells through macropinocytosis followed by endosome trafficking catalyzed by PIK3C3, with the HOPS complex also implicated in this process. Following internalization, the virion-SLC39A9 complex interacts with the cellular receptor NPC1, mediated by SLC39A9’s interaction with the NPC1 CTD domain. After this, the cathepsin B and L cleave viral GP into primed GP (GPcl), exposing the RBD. The exposed RBD then interacts directly with NPC1, promoting viral membrane fusion and releasing viral RNP into cytoplasm (**Fig 8**). However, the precise interaction interface between GP, SLC39A9 and NPC1 requires further investigation, which is crucial for the development of potential antiviral compounds.

In addition, SLC39A9 has been identified as a glycan-regulating factor through post-transcriptional regulation of glycosyltransferases and plays a role in the secretory pathway [86]. This raises the possibility that SLC39A9 might affect the glycosylation of the viral GP, thus influencing viral assembly. However, our VLP assembly assays showed no significant difference in VLP production or GP incorporation between WT and SLC39A9 KO cells (**Figs 3G–3I and S4C**), while only EBOVΔVP30-EGFP entry was significantly impaired (**Fig 3A and 3B**). Given that the GP protein of EBOV is glycosylated, which can impact viral entry [87–91]. It is worthwhile to investigate the indirect role of SLC39A9 in the glycosylation of GP and its implications for the EBOV life cycle, in addition to its direct function in facilitating GP and NPC1 interaction. Furthermore, It was reported that the effects of SLC39A9 on N-glycosylation is dependent on C1GalT1 (Core 1 synthase, glycoprotein-N-acetylgalactosamine 3-beta-galactosyltransferase, 1), which is responsible for synthesis of Core 1 by the transfer of Gal from UDP-Gal to GalNAc-alpha-1-R [92–94]. Notably, unlike the phenotype caused by SLC39A9 knockout, we found that C1GalT1 knockout in Huh7.5.1-VP30 cells did not impair EBOVΔVP30-EGFP infection (**Fig 2A**), indicating that SLC39A9-mediated glycosylation has limited effect on EBOV infection. Still, more detailed assays are needed to clarify the effect of SLC39A9 on virion GP incorporation and/or viral entry if viral GP glycosylation was affected. This could be addressed by dissecting GP glycosylation on virions produced in WT or SLC39A9 KO cells and challenging cells with replication-defective VLPs produced in WT or KO cells in future studies.

Moreover, our screening results revealed the essential role of ASGR1 and ASGR2 in facilitating sufficient EBOV infection (**Figs 1 and 2A**). Previous studies have underscored their significance in Marburg virus infection [95]. It would be worthwhile to investigate their functionality in various filoviruses and distinct Ebola virus strains, such as the Sudan virus, which still lacks effective treatments [96]. Notably, they have also been implicated as entry factors to SARS-CoV2 [97] and liver related viruses such as HEV [98], HBV [99] and HCV [100], suggesting a broad-spectrum involvement in viral infections. Their potential as host targets for developing broad-spectrum antiviral compounds is particularly noteworthy.

It’s important to acknowledge the limitations of our study. Firstly, we lack evidence of the interaction between EBOV GP and endogenous SLC39A9 due to the unavailability of a reliable antibody recognizing endogenous SLC39A9. Secondly, the functional roles attributed to PIK3C3 and SLC39A9 in EBOV infection are primarily based on viral infection assays conducted in vitro using genetic knockout cells, where the efficiency of knockout varied. Thus, future studies could consider using genetic knockout mouse models to provide a more comprehensive understanding of the roles of SLC39A9 and PIK3C3 in EBOV infection *in vivo*, offering insights beyond the cellular context. In summary, our study has unveiled critical factors essential for EBOV infection, with a specific focus on two novel factors, PIK3C3 and SLC39A9, which play pivotal roles in efficient EBOV entry through distinct mechanisms. The identification of these novel critical host factors provides compelling candidates for the development of pharmacological interventions against EBOV infection.

## Materials and methods

### Cell culture and viruses

HEK293T cells, Huh7 cells, Huh7.5.1 cells and Huh7/Huh7.5.1-VP30 previously constructed [35] were cultured in Dulbecco’s modified Eagle medium (Gibco) supplemented with 10% fetal bovine serum, and 50 IU/mL penicillin/streptomycin, and cells were maintained at 37°C and 5% CO_2_. Regular testing ensured that all cell lines used were free of mycoplasma contamination. The following viruses were used: EBOVΔVP30-EGFP virus was rescued and amplified as previously described [35]. Authentic Ebola virus (Mayinga strain) experiments were conducted in a Biosafety Level 4 (BSL-4) laboratory.

### Chemicals

Zinc Chelator TPEN (Tetrakis-(2-pyridylmethyl) ethylenediamine) (#HY-100202), Zinc ionophore Zinc Pyrithione (ZnPy, # HY-B0572) and PIK3C3 inhibitor VPS34-IN-1(#HY-12795) were was obtained from MedChemExpress (MCE).

### Lentivirus production and transduction

Basic procedure is same as described [101]. Lentivirus supernatants were collected at 36, 60 and 84 h after transfection, pooled, passed through a 0.45-μm filter, aliquoted, and frozen at -80°C. For lentiviral transduction, cells were seeded and infected at the following 2 days with lentiviruses.

### Genome-wide CRISPR/Cas9 KO screen

Human whole-genome sgRNA library Brunello in backbone lentiGuide-Puro targeting 19114 genes and containing 76441 unique sgRNAs along with 1000 non-targeting controls (Addgene, #73178) [37,40]. The library was packaged and titrated with the above-described lentivirus package system, 48h and 72h post transfection, supernatants were collected, filtered and aliquoted for storage at -80°C. For the screen, Huh7.5.1-VP30 cells were stably transduced with lentivirus from lentiCas9-Blast (Addgene, #52962) and subsequently selected using blasticidin. Huh7.5.1-VP30-Cas9 cells were transduced with lentiviral sgRNA library at a multiplicity of infection (MOI) of 0.3 and selected with puromycin and expanded for 7 days. 4×10^7^ surviving cells (average 500-fold coverage of the library) were infected with EBOVΔVP30-EGFP virus at MOI = 1, In parallel, same number of cells were seeded as uninfected input samples. The experiments were performed in parallel as triplicate biological replicates. 3 days post infection, uninfected GFP negative cells were sorted with FACS. Sorted cells were expanded and challenged with EBOVΔVP30-EGFP again at the same MOI. 3dpi, sorting virus resistant cells again. A third challenge and sorting were done following same procedure. Genomic DNA was extracted from the uninfected cells harvested on the same day of first sorting and the resistant cells that survived in each round of virus infection. sgRNA sequences were then amplified by ExTaq DNA Polymerase (Clontech) and purified with Agencourt AMPure XP SPRI beads according to manufacturer’s instructions (Beckman Coulter, A63880). Samples were then subjected to next-generation-sequencing on an Illumina HiSeq X Ten (Annoroad).

### Generation of gene-specific KO cell lines

For validation of screen hits, the two best individual sgRNAs (**S3 Table**) and two Non-Targeting-Control sgRNAs (NTCs) were picked from the sgRNA library Brunello and Huh7/Huh7-VP30/Huh7.5.1-VP30, 293T KO cell lines were generated following the standard protocol [37,39].

### Authentic EBOV infection experiments

Experiments using live infectious EBOV (Zaire Mayinga strain) were performed in BSL-4 facilities at Wuhan National Biosafety Laboratory, following approved standard operating procedures. Huh7 WT or KO cells were infected with authentic EBOV at a MOI = 0.1. At 3 days post infection, cells were collected and treated with TRIzol to purify the RNA for RT-qPCR assay.

### RNA isolation and RT-PCR

Basic procedure is same as described [102]. Primers used in qPCR are as follows: EBOV NP: 5’-CCG TTC AAC AGG GGA TTG TTC G-3’ (THU-949) and 5’-CTG CTG GCA GCA ATT CCT CAAG-3’ (THU-950); EBOV 3’UTR: 5’- ACG GTG ATA GCC TTA ATC TTT G -3’ (THU-2133) and 5’- GTA TTT CTG ATT TTA CAG TCC TGC C -3’ (THU-3788); Human GAPDH: 5’-GAA GGT GAA GGT CGG AGT C-3’ (THU-003) and 5’-GAA GGT GAA GGT CGG AGT C-3’ (THU-004); NPC1: 5’- CAG AAC AGC CAT TCC GTG CTG -3’ (THU-4582) and 5’- CTG GTC CAC CAA ACG TAC CCA G -3’ (THU-6854); MT1A: 5’-AGA GTG CAA ATG CAC CTC CTG C-3’ (THU5626) and 5’-CGG ACA TCA GGC ACA GCA GCT-3’ (THU5627); MT2A: 5’-GGC TCC TGC AAA TGC AAA GAG TG-3’ (THU5628) and 5’-AGC AGC TGC ACT TGT CCG AC-3’ (THU5629). The thermal cycling protocol was as follows: 95°C for 5 min followed by 40 cycles consisting of 95°C for 15 s, 60°C for 1 min. Comparison of RNA expression levels between samples was performed according to the ΔΔCT method with GAPDH as internal control.

### Western blotting assay

Basic procedure is same as described [103]. The blots were exposed to the primary antibodies mouse anti-Flag (Sigma, #F1804) mouse anti-HA (Abmart, #26D11) rabbit anti-NPC1(Abclonal, #A4795), rabbit anti-PIK3C3(CST, #4263S), rabbit anti-SLC39A9 (Sangon, #D162239) rabbit anti-NP, and mouse anti-β-tubulin (CWBIO, #CW0098) in 5% nonfat milk in 1× PBS containing 0.1% Tween 20 for 2 h. The blots were then washed in 1× PBS containing 0.1% Tween 20. After 1 h exposure to HRP-conjugated secondary antibodies (Abclonal), the blots were washed again before using the luminescent image analyzer ImageQuant LAS 4000 (GE Healthcare).

### Immunoprecipitation assay

For each sample, about 2×10^6^ cells were washed with ice-cold PBS and lysed in 440 μL Protein Lysis Buffer (same as WB assay) on ice for 30 min. Lysates were centrifuged at 12000rpm at 4C for 15min, and supernatant was transferred to new tubes. 40 μL was saved as input sample. For immunoprecipitation of Flag-tagged proteins, lysates were incubated with 5 μL Anti-Flag M2 magnetic beads (Sigma) 5-8h at 4°C while rotating. Beads were washed 5 times with lysis buffer and proteins were eluted using 40 μL of 0.3 mg/ml Flag peptide (GL Biochem) for 30 min on ice. Eluates were mixed with 10 μL 5×SDS-Loading. For immunoprecipitation of HA-tagged proteins, lysates were incubated with 5 μL Anti-HA-Nanoab-Magnetic beads (Lablead) for 5-8h at 4°C while rotating. Beads were washed 5 times with lysis buffer and proteins were eluted using 50 μL 1×SDS-Loading. Samples were analyzed by western blot as described above.

### Confocal immunofluorescence microscopy

Huh7.5.1-VP30 cells infected by EBOVΔVP30-EGFP virus or mCherry-VLPs in confocal dishes were fixed with 4% PFA (DF0135, Leagene) at room temperature for 15 min and wash with PBS for three times. EBOVΔVP30-EGFP infected cells were staining with Human anti-GP Ab (purified EBOV neutralizing Ab206 [65], mouse anti-Flag (Sigma), mouse anti-HA (Abmart, #26D11) as described and corresponding fluorescence Secondary Ab: Alexa-fluor568/488/647 Goat anti Human/Mouse IgG (Invitrogen). Nucleus was stained with 1 μg/ml DAPI for 10 min. Images were collected under Zeiss LSM880 with Airyscan confocal laser scanning microscope using a 63× objective lens. For GP puncta quantification, optical sections perpendicular to the z axis were performed with the optimal interval throughout the sample. Thirteen to twenty-two z-stacks were acquired per image. The confocal pictures were reconstructed by projection of sections.

### Viral attachment and entry assay

WT, NTC (Non-Targeting Control), NPC1-KO, PIK3C3-KO or SLC39A9-KO Huh7 cells were inoculated with EBOVΔVP30-EGFP at an MOI of 10–20. For VLP entry assay, 20ul of concentrated mCherry-VLP (WT or F535R) was used. For viral attachment and entry assay, cells were incubated for 1h on ice or 4h in 37°C incubator respectively. At each harvest time point, cells were washed three times with PBS and processed for RT-qPCR or immunofluorescence as described above.

### EBOV minigenome replicon assay

The Huh7-4P cell line and minigenome replicon construct containing a Gluc and hygromycin selection marker were gifts from Prof. Jin Zhong [48]. *In vitro* transcribed replicon RNA (#TR102, Vazyme) was electroporated into Huh7-4P cells followed by hygromycin selection for one week.

### EBOV Virus-like-particle production

EBOV virus-like-particles (VLPs) were produced by co-transfecting HEK293T cells with pcDNA3 plasmids encoding EBOV NP, flag-tagged EBOV VP40 or mCherry-VP40 and EBOV WT GP-HA or F535R mutant using PEI transfection reagent at a 1:1:1 ratio. Virus-containing supernatants were collected and concentrated by ultracentrifugation (100,000g, 4°C, 2h; Beckman Coulter Optima XPN-100, SW32Ti rotor) through a 20% (wt/vol) sucrose cushion. Viruses were resuspended in phosphate buffered saline (PBS) and stored at -80°C before analyzing with WB assay.

### Membrane protein detection with biotin labeling assay

SLC39A9 and ACE2 transfected confluent HEK293T cells (2 days) were pre-chilled on ice for 10min and wash twice with PBS [pH 7.4] supplemented with 1.5 mM MgCl_2_, 0.2 mM CaCl_2_); 2 mg/ml NHS-SS-Biotin in PBS was supplemented on cells to label cell surface proteins at 4°C for 30min. unbound biotin was washed and quenched with 100mM glycine for 5 min, three times. Labeled cells were subjected to IP assay after wash twice with PBS.

### Protein purification

The EBOV transmembrane-and-mucin-domains-removed GP (GPΔMUC) protein was constructed into pCAG expression vector as previously described [104] and expressed in mammalian expression system using 293FT cells. Soluble GPΔMUC protein tagged with 6×His tag was harvested and purified from the culture supernatants by Ni-NTA agarose (Qiagen, #30210) gel chromatography. To mimic endosomal protease cleavage and produce primed EBOV GP (GPcl), which was capable of NPC1 binding, 2 mg of EBOV GPΔMUC protein was incubated with 500 μg/ml thermolysin (Sigma) at 37°C for 2h and purified on a Superdex 200 Increase column. The NPC1-10×His-Flag protein was purified following protocol as described before [68]. The SLC39A9-6×His-2×Strep was constructed into pCAG vector and expressed in 293FT cells. 72h posts transfection, cells were lysed with 1%DDM containing Protein Lysis Buffer and purified with Strep-Tactin agarose resin (IBA, #2-1208-010) and gel filtration with Superdex 200 Increase in the AKTA Purifer100.

### *In vitro* pull-down assay

10 μg of two indicated proteins were incubated together with 5 μL Flag M2 magnetic beads or Strep-Tactin agarose beads at a 500 μL system (25mM Tris, 150mM NaCl, 0.01%DDM, pH = 8, supplemented with 1mM PMSF) by rotating at 4°C for 2~4h. Beads were washed five times with incubation buffer and collected with magnetic frame. Bound proteins were eluted with 1×SDS-loading and further subjected to SDS-PAGE and WB analysis.

### Infection block assay

EBOVΔVP30-EGFP virus was pre-incubated with different doses of SLC39A9 proteins or an unrelated VZV gE protein or EBOV neutralizing Ab Q206 for 1h at 37°C before inoculated to Huh7.5.1-VP30 cells (MOI = 0.1). Two days post infection, virus infection was detected by flow cytometry to quantify EGFP positive cells.

### Thermolysin treatment of EBOVΔVP30-EGFP

EBOVΔVP30-EGFP virus was treated with thermolysin (500μg/ml) at 37° for 2h. Subsequently, the digested and untreated viruses were concentrated through 20% sucrose by centrifugation at 100,000g for 2h. The cleavage of the virion GP was confirmed by Western blot analysis.

### Surface plasmon resonance analysis

NPC1, SLC39A9 was immobilized on a CM5 chip (GE Healthcare) to a level of around 2500 and 500 response units respectively using a Biacore 8K plus (GE Healthcare) and a running buffer (10 mM HEPES pH 7.2, 150 mM NaCl and 0.02% GDN). Serial dilutions of the SLC39A9, GP ΔMUC, GPcl were flowed over the chip surface. The resulting data were fit to a 1:1 binding model using Biacore Evaluation Software (GE Healthcare).

### Statistical analysis

Unless otherwise mentioned, results are represented as means ± standard deviations determined using GraphPad Prism 8 (GraphPad Software, La Jolla, CA). One-way analysis of variance (ANOVA) with Tukey’s honestly significant difference (HSD) test was used to assess statistical significance of the differences between the different group parameters. *p* values of less than 0.05 were considered statistically significant.

## Supporting information

S1 Fig Increased virus resistance during CRISPR/Cas9 KO screen. Huh7.5.1-VP30-Cas9 library cells were infected with EBOVΔVP30-EGFP virus (MOI = 1) for 3 days. Virus resistant cells (GFP negative) were sorted by FACS. Three rounds of infection and sorting were performed. Samples for sequencing were harvested at every sorting. The infection results were recorded (left) and cell states were observed on fluorescent microscope (right). Each selection was performed with three biological replicates. Scale bar, 100 μm.
(TIF)

S2 Fig **(A)** EBOVΔVP30-EGFP virus infected WT or KO Huh7.5.1-VP30 cells (MOI = 1, 2dpi) were analyzed by flow cytometry. Experiments were independently repeated three times with similar results. Representative results were shown. Quantification results were shown in **Fig 2. (B)** Huh7.5.1-VP30 cells with sgNC or knockout of PIK3C3 SLC39A9 were exposed to EBOVΔVP30-EGFP, VSV-Venus or HSV-GFP viruses. Viral infection levels was quantified using FACS. Data were normalized to sgNC control. Error bars indicate mean ± SEM. Significance was assessed by one-way ANOVA, with the asterisks denoting significant differences: ns, no significance (p ≥ 0.05); *, p < 0.05; **, p< 0.01; ***, p< 0.001.
(TIF)

S3 Fig NPC1 mRNA and protein level detection. NTC, PIK3C3 or SLC39A9 KO Huh7.5.1-VP30 cells were harvested for RT-qPCR analysis targeting NPC1 mRNA **(A)** and lysed for WB detection of NPC1 protein level **(B).**
(TIF)

S4 Fig EBOV life cycle dissection. Related to **Fig 3**. **(A)** EBOV minigenome replicon RNA was *in vitro* transcribed and electroporated into Huh7-4P cells; Hygromycin selected replicon cells were treated with different doses of remdesivir. Viral RNA level was quantified by RT-qPCR at 3 days after drug treatment **(B)**. **(C)** Quantification of EBOV NP and GP in EBOV VLPs from **Fig 3I**. Intensity quantification was done with ImageJ. Error bars represent mean ± SD. Significance assessed by one-way ANOVA, the asterisks represent significant differences: ns, no significance (p ≥ 0.05); *, p < 0.05; **, p< 0.01; ***, p< 0.001.
(TIF)

S5 Fig MT1A and MT2A mRNA level detection. Huh7.5.1-VP30 cells were treated with **(A)**TPEN or **(B)** ZnPy for 4h or 2d at indicated concentrations before RNA extraction. MT1A and MT2A mRNA level were then quantified by RT-qPCR. mRNA level was represented as normalization to GAPDH.
(TIF)

S6 Fig Membrane SLC39A9 protein detection and protein purification. **(A)** Membrane SLC39A9 protein detection with biotin labeling assay. SLC39A9-Flag and ACE2 were co-transfected into HEK293T cells for 2 days. Transfected cells were then labeled with NHS-SS-biotin and lysates were further subjected to IP with Strep-Tactin resin and WB analysis. **(B)** Diagram of SLC39A9 protein construct for purification. 6×his and 2×Strep tag was added in the C-terminal of SLC39A9 mRNA sequence. **(C)** Diagram of EBOV GP protein and C-terminal 6×his tagged GPΔMUC construct for purification. **(D)** Purification of SLC39A9 through size exclusion chromatography. The indicated elute fractions were applied to SDS-PAGE and visualized by Coomassie blue staining. Red asterisk, SLC39A9 proteins. **(E)** GPΔMUC -6×his was purified with Ni-NTA agarose beads and elute with 300 mM imidazole. Elutes were sampled for SDS-PAGE analysis and further validation by WB w/wo PNGase treatment **(F)**.
(TIF)

S7 Fig Intracellular membrane SLC39A9 detection and NPC1 protein purification. **(A)** SLC39A9-HA and NPC1-EGFP stably transduced Huh7 cells were transfected with RFP fused lysosome marker (LAMP1), Golgi marker (GS28) or ER marker (Calreticulin ER signal peptide) and stained with DAPI for confocal analysis. Pictures were captured on LSM880 (Carl Zeiss). Representative figures were shown out of at least five different views with similar pattern. **(B)** Diagram and purification of NPC1 protein. C terminal of NPC1 was tagged with Flag and his. Purification of full-length hNPC1 through size exclusion chromatography, indicated fractions were applied to SDS-PAGE and visualized by Coomassie blue staining.
(TIF)

S8 Fig Interaction detection under virus infection. **(A)** SLC39A9-Flag or **(B)** NPC1-Flag expressed Huh7-VP30 cells were infected with EBOVΔVP30-EGFP virus (MOI = 1, 2dpi) and cell lysates were then harvested for WB analysis with indicated Abs. Tubulin was included as an internal control. This experiment was repeated for three times with similar results.
(TIF)

S9 Fig *In vitro* interaction detection of SLC39A9, EBOV GP and NPC1. **(A)** Purified GPΔMUC-his protein (from **S4G Fig**) was catalyzed with 500 μg/ml thermolysin at 37°C for 2h and **(D)** purified by size exclusion chromatography. **(C)** Indicated fractions were then analyzed by SDS-PAGE and Coomassie staining. **(D)** BIAcore diagram of purified GPcl bound to NPC1 protein. SPR assays were independently repeated twice with similar results. **(E)** Pull-down assay. 10 μg purified SLC39A9-Flag and GPΔMUC-His or GPcl proteins were incubated at 4°C for 4h together with Strep-Tactin agarose beads before immunoblotting. **(F)** EBOVΔVP30-EGFP was treated with or without thermolysin (500 μg/ml) at 37°C for 2h. Cleavage of EBOVΔVP30-EGFP GP was verified by Western blot analysis. **(G)** Huh7.5.1 NC or SLC39A9 KO cells were infected with EBOVΔVP30-EGFP (EBOV) or thermolysin-cleaved EBOVΔVP30-EGFP (EBOVcl) for 2 days. Viral infection was assessed by quantifying the rate of GFP-positive cells using FACS.
(TIF)

S1 Table The top 300 ranked gene list of screens. (XLSX)

S2 Table Gene list of GO analysis and detailed GO terms. (XLSX)

S3 Table Targeted individual sgRNA and primer sequences. (XLSX)

S1 Data Raw data corresponding to Figs 1–7 are compiled here. (ZIP)
