# Supplementary figures and images for: Genome-wide CRISPR/Cas9 screen identifies SLC39A9 and PIK3C3 as crucial entry factors for Ebola virus infection
Source: PLoS Pathog. 2024 Aug 22;20(8):e1012444. doi: 10.1371/journal.ppat.1012444 (PMC11341029; doi:10.1371/journal.ppat.1012444)

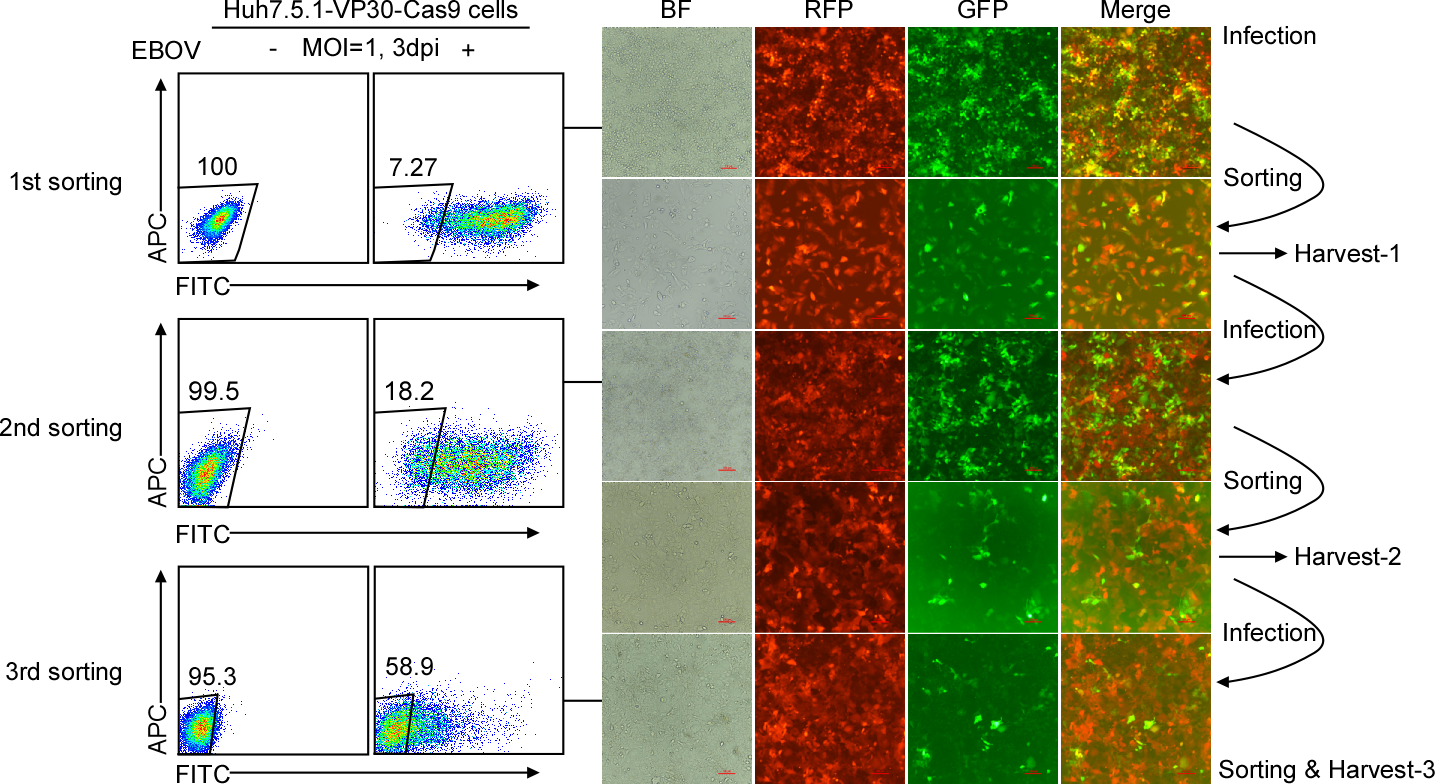

Supplement: S1 Fig — Huh7.5.1-VP30-Cas9 library cells were infected with EBOVΔVP30-EGFP virus (MOI = 1) for 3 days. Virus resistant cells (GFP negative) were sorted by FACS. Three rounds of infection and sorting were performed. Samples for sequencing were harvested at every sorting. The infection results were recorded (left) and cell states were observed on fluorescent microscope (right). Each selection was performed with three biological replicates. Scale bar, 100 μm. (TIF) [file ppat.1012444.s001.tif]

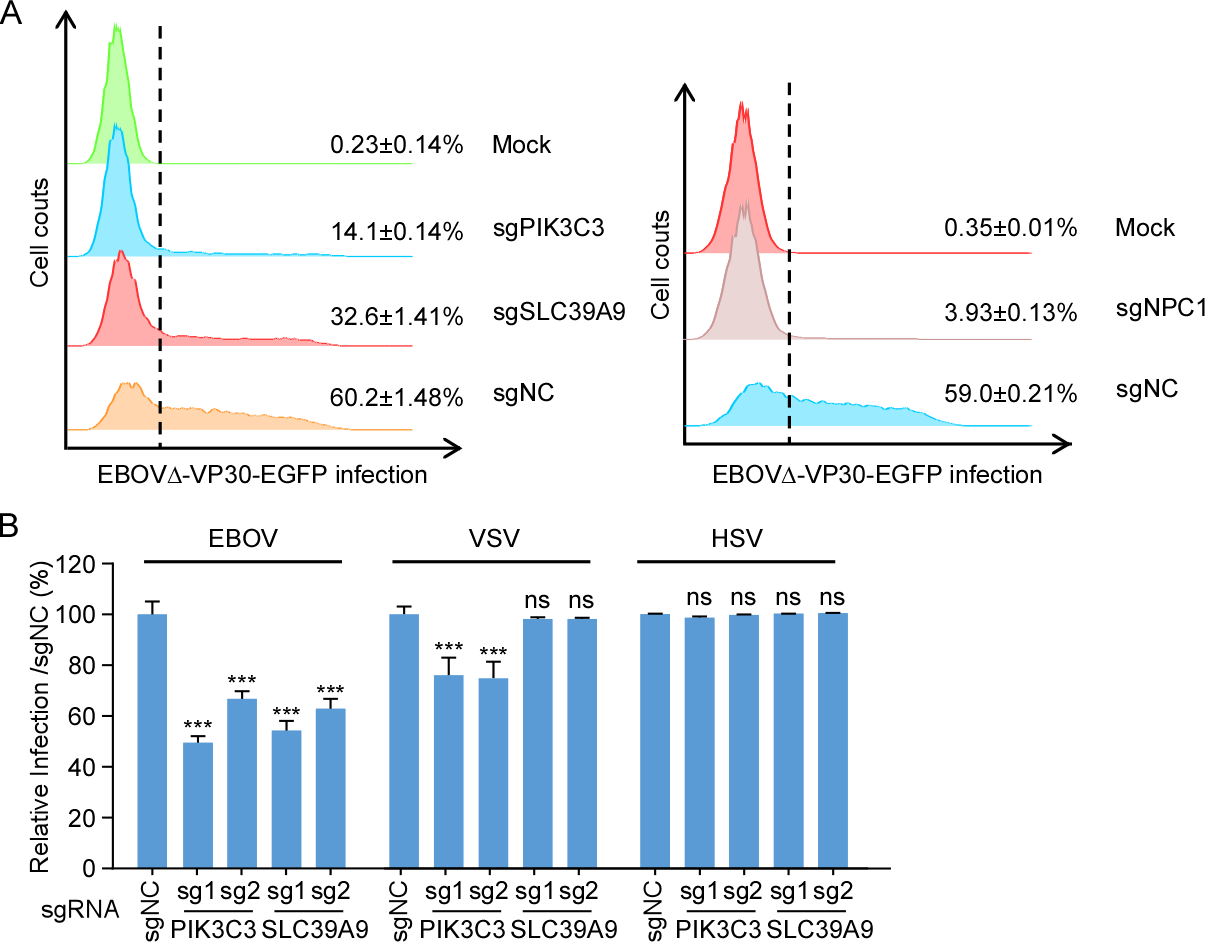

Supplement: S2 Fig — (A) EBOVΔVP30-EGFP virus infected WT or KO Huh7.5.1-VP30 cells (MOI = 1, 2dpi) were analyzed by flow cytometry. Experiments were independently repeated three times with similar results. Representative results were shown. Quantification results were shown in Fig 2. (B) Huh7.5.1-VP30 cells with sgNC or knockout of PIK3C3 SLC39A9 were exposed to EBOVΔVP30-EGFP, VSV-Venus or HSV-GFP viruses. Viral infection levels was quantified using FACS. Data were normalized to sgNC control. Error bars indicate mean ± SEM. Significance was assessed by one-way ANOVA, with the asterisks denoting significant differences: ns, no significance (p ≥ 0.05); *, p < 0.05; **, p< 0.01; ***, p< 0.001. (TIF) [file ppat.1012444.s002.tif]

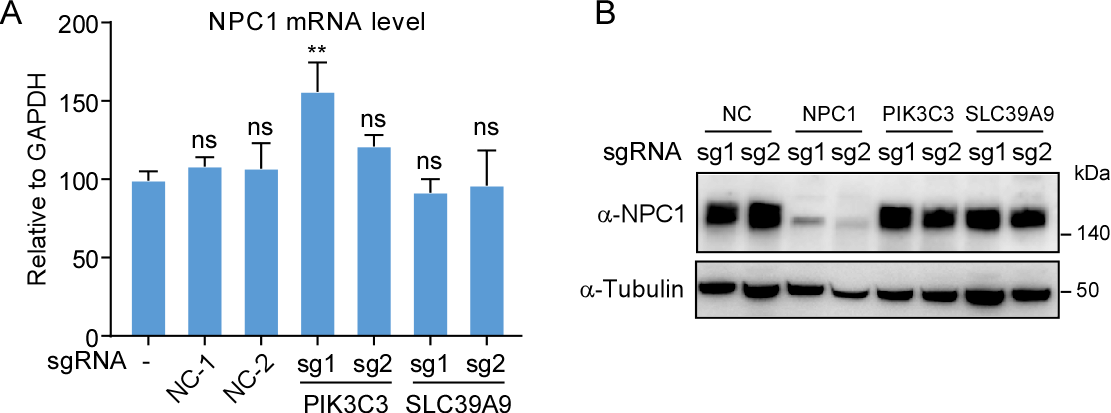

Supplement: S3 Fig — NTC, PIK3C3 or SLC39A9 KO Huh7.5.1-VP30 cells were harvested for RT-qPCR analysis targeting NPC1 mRNA (A) and lysed for WB detection of NPC1 protein level (B). (TIF) [file ppat.1012444.s003.tif]

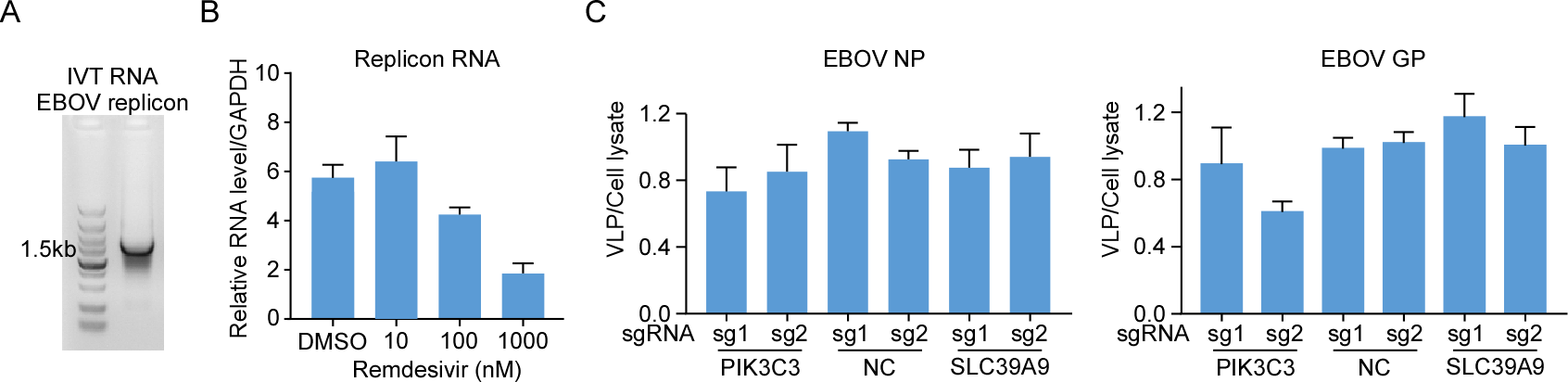

Supplement: S4 Fig — Related to Fig 3. (A) EBOV minigenome replicon RNA was in vitro transcribed and electroporated into Huh7-4P cells; Hygromycin selected replicon cells were treated with different doses of remdesivir. Viral RNA level was quantified by RT-qPCR at 3 days after drug treatment (B). (C) Quantification of EBOV NP and GP in EBOV VLPs from Fig 3I. Intensity quantification was done with ImageJ. Error bars represent mean ± SD. Significance assessed by one-way ANOVA, the asterisks represent significant differences: ns, no significance (p ≥ 0.05); *, p < 0.05; **, p< 0.01; ***, p< 0.001. (TIF) [file ppat.1012444.s004.tif]

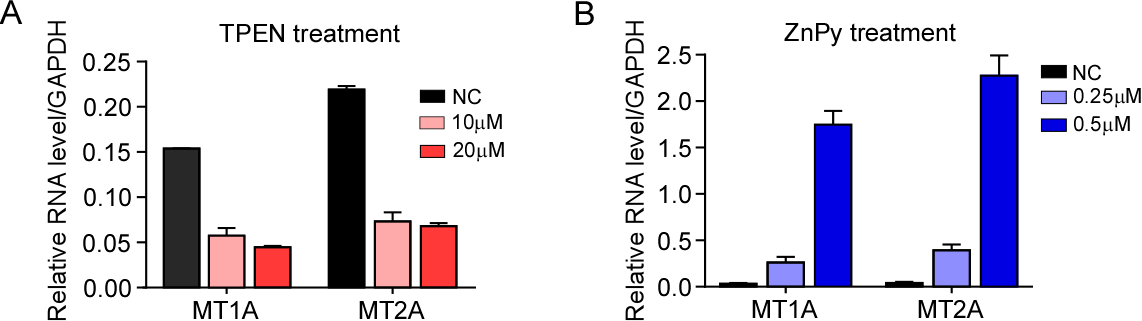

Supplement: S5 Fig — Huh7.5.1-VP30 cells were treated with (A)TPEN or (B) ZnPy for 4h or 2d at indicated concentrations before RNA extraction. MT1A and MT2A mRNA level were then quantified by RT-qPCR. mRNA level was represented as normalization to GAPDH. (TIF) [file ppat.1012444.s005.tif]

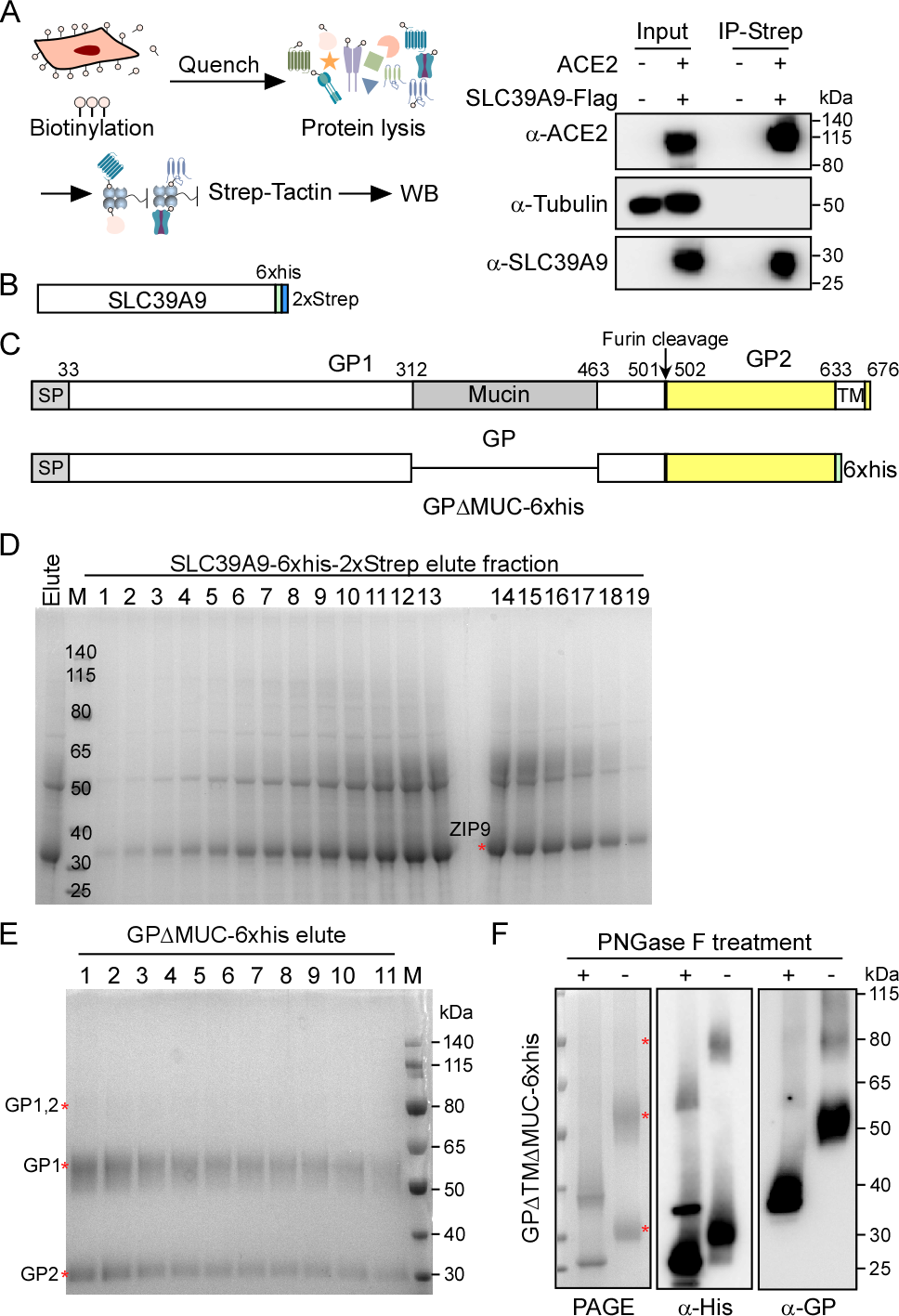

Supplement: S6 Fig — (A) Membrane SLC39A9 protein detection with biotin labeling assay. SLC39A9-Flag and ACE2 were co-transfected into HEK293T cells for 2 days. Transfected cells were then labeled with NHS-SS-biotin and lysates were further subjected to IP with Strep-Tactin resin and WB analysis. (B) Diagram of SLC39A9 protein construct for purification. 6×his and 2×Strep tag was added in the C-terminal of SLC39A9 mRNA sequence. (C) Diagram of EBOV GP protein and C-terminal 6×his tagged GPΔMUC construct for purification. (D) Purification of SLC39A9 through size exclusion chromatography. The indicated elute fractions were applied to SDS-PAGE and visualized by Coomassie blue staining. Red asterisk, SLC39A9 proteins. (E) GPΔMUC -6×his was purified with Ni-NTA agarose beads and elute with 300 mM imidazole. Elutes were sampled for SDS-PAGE analysis and further validation by WB w/wo PNGase treatment (F). (TIF) [file ppat.1012444.s006.tif]

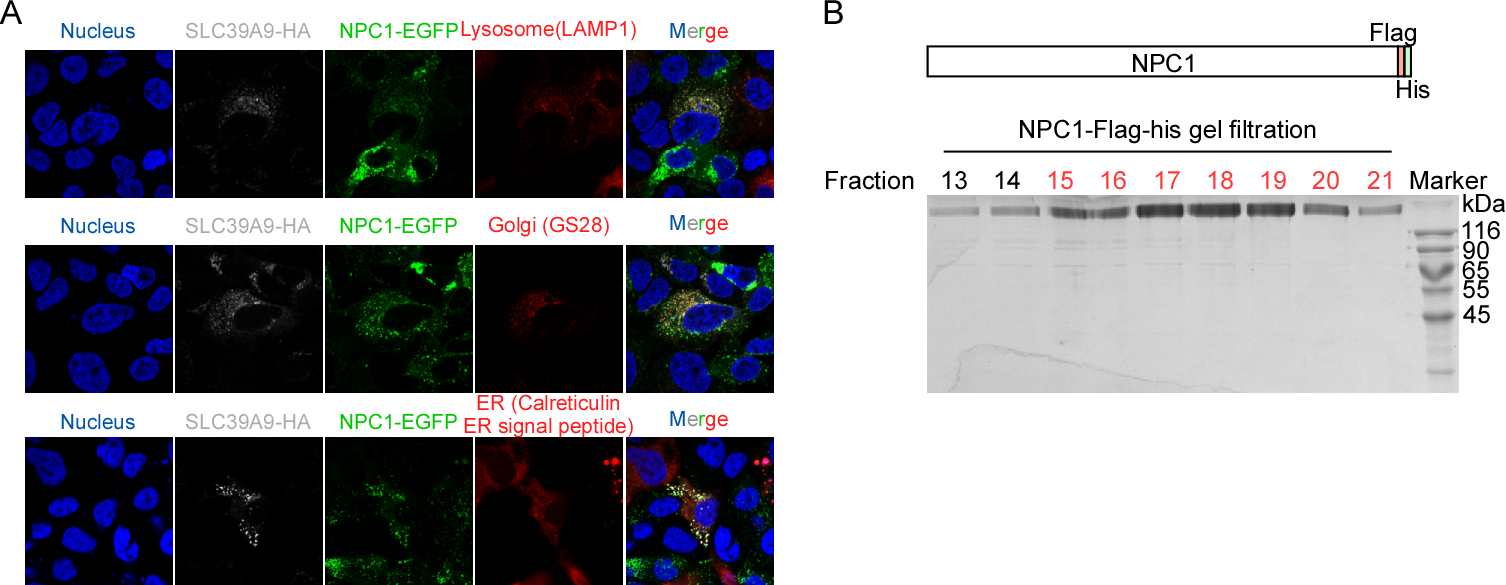

Supplement: S7 Fig — (A) SLC39A9-HA and NPC1-EGFP stably transduced Huh7 cells were transfected with RFP fused lysosome marker (LAMP1), Golgi marker (GS28) or ER marker (Calreticulin ER signal peptide) and stained with DAPI for confocal analysis. Pictures were captured on LSM880 (Carl Zeiss). Representative figures were shown out of at least five different views with similar pattern. (B) Diagram and purification of NPC1 protein. C terminal of NPC1 was tagged with Flag and his. Purification of full-length hNPC1 through size exclusion chromatography, indicated fractions were applied to SDS-PAGE and visualized by Coomassie blue staining. (TIF) [file ppat.1012444.s007.tif]

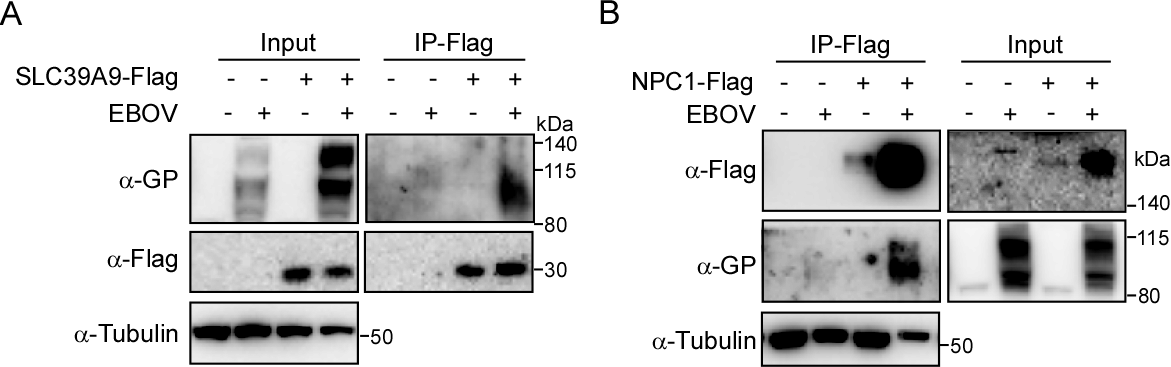

Supplement: S8 Fig — (A) SLC39A9-Flag or (B) NPC1-Flag expressed Huh7-VP30 cells were infected with EBOVΔVP30-EGFP virus (MOI = 1, 2dpi) and cell lysates were then harvested for WB analysis with indicated Abs. Tubulin was included as an internal control. This experiment was repeated for three times with similar results. (TIF) [file ppat.1012444.s008.tif]

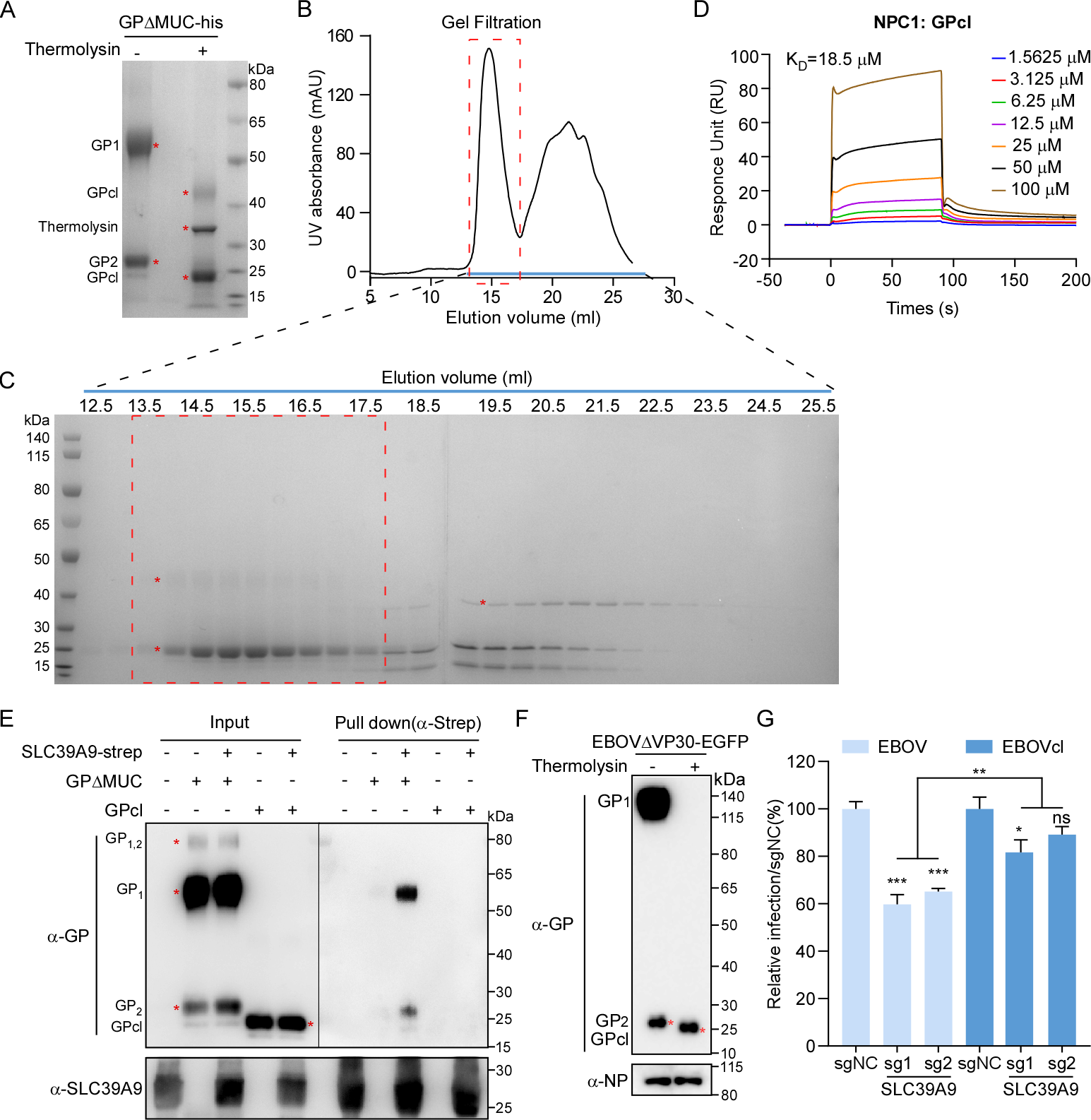

Supplement: S9 Fig — (A) Purified GPΔMUC-his protein (from S4G Fig) was catalyzed with 500 μg/ml thermolysin at 37°C for 2h and (D) purified by size exclusion chromatography. (C) Indicated fractions were then analyzed by SDS-PAGE and Coomassie staining. (D) BIAcore diagram of purified GPcl bound to NPC1 protein. SPR assays were independently repeated twice with similar results. (E) Pull-down assay. 10 μg purified SLC39A9-Flag and GPΔMUC-His or GPcl proteins were incubated at 4°C for 4h together with Strep-Tactin agarose beads before immunoblotting. (F) EBOVΔVP30-EGFP was treated with or without thermolysin (500 μg/ml) at 37°C for 2h. Cleavage of EBOVΔVP30-EGFP GP was verified by Western blot analysis. (G) Huh7.5.1 NC or SLC39A9 KO cells were infected with EBOVΔVP30-EGFP (EBOV) or thermolysin-cleaved EBOVΔVP30-EGFP (EBOVcl) for 2 days. Viral infection was assessed by quantifying the rate of GFP-positive cells using FACS. (TIF) [file ppat.1012444.s009.tif]
